# Integrative analysis of cerebrospinal fluid biomarkers, metabolomics, and polygenic risk reveals novel metabolite associations with Alzheimer's disease

**DOI:** 10.1177/13872877251389924

**Published:** 2025-11-07

**Authors:** Marcelo Francia, Naren Ramesh, Toni Boltz, Merel Bot, Wiesje M van der Flier, Pieter Jelle Visser, Sven van der Lee, Charlotte E Teunissen, Yolande AL Pijnenburg, Anouk den Braber, Loes Olde Loohuis, Lianne M Reus, Betty M Tijms, Roel A Ophoff

**Affiliations:** 1 Center for Neurobehavioral Genetics, Semel Institute for Neuroscience and Human Behavior, University of California, Los Angeles, CA, USA; 2 Interdepartmental Program for Neuroscience, David Geffen School of Medicine, University of California Los Angeles, Los Angeles, CA, USA; 3 Department of Human Genetics, David Geffen School of Medicine, University of California Los Angeles, Los Angeles, CA, USA; 4 Alzheimer Center Amsterdam, Neurology, Vrije Universiteit Amsterdam, Amsterdam UMC location VUmc, Amsterdam, The Netherlands; 5 Amsterdam Neuroscience, Neurodegeneration, Amsterdam, The Netherlands; 6 Department of Psychiatry, Maastricht University, Maastricht, The Netherlands; 7 Department of Neurobiology, Care Sciences and Society, Division of Neurogeriatrics, Karolinska Institutet, Stockholm, Sweden; 8 Section Genomics of Neurodegenerative Diseases and Aging, Department of Human Genetics, Vrije Universiteit Amsterdam, Amsterdam UMC, Amsterdam, the Netherlands; 9 Neurochemistry Lab and Biobank, Department of Clinical Chemistry, Amsterdam Neuroscience, Amsterdam UMC, Amsterdam, The Netherlands; 10 Department of Biological Psychology, Amsterdam Public Health Research Institute, Vrije Universiteit Amsterdam, Amsterdam, The Netherlands

**Keywords:** Alzheimer's disease, amyloid-beta, biomarkers, cerebrospinal fluid, genetics, metabolomics, tau

## Abstract

**Background:**

Cerebrospinal fluid (CSF) metabolomics offers an opportunity to investigate *in vivo* biological pathways impacted in the human brain by Alzheimer's disease (AD). While impairments in brain glucose metabolism and lipid homeostasis are implicated in AD, the underlying metabolic pathways remain unclear. Genotype information can also be leveraged to study associations between CSF metabolites and AD genetic risk.

**Objective:**

To evaluate how CSF metabolomic profiles and genetic risk are associated with AD pathology as reflected by established CSF biomarkers (Aβ, P-Tau, and T-Tau).

**Methods:**

We collected CSF mass spectrometry measurements of 678 metabolites and 4865 unnamed compounds, as well as genome-wide genotype data from 487 individuals in the Amsterdam Dementia cohort. Polygenic risk scores (PRS) for AD were calculated. Elastic net regression models were used to predict AD biomarker levels with CSF metabolites, and pathway enrichment analysis was performed to assess the metabolic pathways involved.

**Results:**

98 CSF metabolites were found to be significantly correlated with P-Tau or T-Tau, but none with Aβ CSF levels. Elastic net regression models identified 42 and 34 metabolites predicting P-Tau and T-Tau, respectively, including novel associations with Anserine and Fucose. Pathway enrichment analysis implicated Pentose and Glucuronate Interconversions, Glycerophospholipid Metabolism, and ABC Transporters in AD pathology. PRS analysis highlighted four CSF phosphatidylcholines significantly associated with AD genetic risk.

**Conclusions:**

CSF metabolites demonstrate a lack of Aβ levels associations, contrasting with multiple significant findings for P-Tau and T-Tau. Novel associations with Anserine and Fucose may provide new insights into metabolic pathways impacted by AD pathology.

## Introduction

Cerebrospinal fluid (CSF) has emerged as a valuable source for studying ongoing neurobiological processes due to its proximity to the brain. Biomarkers for Alzheimer's disease (AD), such as decreased amyloid-β (Aβ) 1-42 levels and increased levels of phosphorylated tau (P-Tau) and total tau (T-Tau),^1^ can be measured from CSF *in vivo*. These biomarkers are associated with disease status and are used for diagnostic purposes,^2^ reflecting the presence of extracellular Aβ plaques, intracellular tau tangles, and neurodegeneration in AD patients.^3,4^ Metabolic dysfunction has been increasingly implicated in AD pathogenesis, with prior studies linking impairments in glucose metabolism^5^ and lipid homeostasis^6^ to AD. However, the underlying metabolic pathways remain largely unknown. Metabolomics, the comprehensive analysis of thousands of metabolites, offers a powerful tool to study these metabolic pathways and their components simultaneously.^7^ Plasma metabolomics, in particular, has been widely used to examine metabolic alterations associated with AD, revealing disruptions in various biochemical processes. For instance, studies have identified links between AD and alterations in amines and oxidative stress markers,^8^ disruptions in cholesterol and sphingolipid transport,^9^ and abnormalities in ammonia and glutamate metabolism.^10^ Additionally, plasma lipid profiles have been used to predict AD progression,^11,12^ highlighting the potential of metabolomic approaches in understanding disease mechanisms.

Despite these insights, plasma and CSF metabolomics provide distinct perspectives on AD-related metabolic changes. Previous studies have shown that only a subset of metabolites exhibit significant correlations between these biofluids,^13–15^ likely due to the restrictive nature of the blood-brain barrier, which limits molecular exchange between the bloodstream and CSF.^16^ As a result, CSF metabolomics offers a more direct window into metabolic processes occurring within the central nervous system, potentially capturing AD-related changes that may not be reflected in plasma. Previous studies have reported associations between CSF metabolites and P-Tau and T-Tau CSF levels, identifying metabolic pathways involved in tau pathology and neurodegeneration.^15,17,18^ However, these studies did not find a similar pattern for CSF Aβ levels in AD patients. Additionally, these prior studies were limited by small sample sizes (288 individuals) and the number of metabolites tested (326), lacking the ability to provide a comprehensive picture of brain metabolic pathways. A more comprehensive approach, with a larger sample size and a broader range of metabolites, is needed to fully assess the connection between AD CSF biomarkers and *in vivo* metabolic pathways in the human central nervous system.

Metabolites reflect intricate biological processes and hold the potential to gauge the collective impact of a disease, pinpoint biomarkers, and chart disease progression.^17,19^ While metabolite levels are influenced by an individual's physiological state and environmental exposures, it has been shown that genetic factors also play a role. Genome-wide association studies (GWAS) of human blood metabolites have mapped multiple loci influencing the metabolome,^20,21^ with the largest study identifying associations with 690 metabolites at 248 loci.^22^ In the context of CSF, GWAS have shown that CSF metabolite levels are also associated with genetic variants, including those affecting monoamine levels,^23^ amino acids, lipids, and nucleotides.^24–26^ These SNP associations have been used to identify CSF metabolites linked to brain-related phenotypes.^24^ AD is a highly heritable disorder, with the *APOE* ε4 allele being the strongest genetic risk factor.^27,28^ In addition to the *APOE* locus, GWAS of AD have identified more than 80 genetic risk loci, each having a small association with the disease.^29^ The SNP-based heritability of late-onset AD is estimated to be between 36% and 59% in pathologically or CSF-confirmed cases,^30^ with the *APOE* ε4 and *APOE* ε2 alleles explaining up to 25% of the genetic variance.^31^ CSF Aβ, P-Tau, and T-Tau levels are also heritable traits, as underscored by previous GWAS.^4,32^ While the *APOE* locus predominantly predicts Aβ CSF levels, it is also associated with P-Tau and T-Tau levels, even though other genetic loci are more strongly associated with the Tau biomarkers.^4^ Genetic loci associated with AD can be harnessed to construct polygenic risk scores (PRS), which aggregate the genetic effects of these regions to evaluate an individual's susceptibility to AD.^33^ Integrating these PRS measurements with metabolites can help identify metabolic profiles associated with a disease's genetic risk, highlighting potential metabolism regulators intertwined with disease mechanisms. For instance, an atlas of polygenic scores from the UK Biobank identified associations between the PRS for body mass index and coronary heart disease with metabolite levels of lipoproteins.^34^

Our study leveraged AD CSF biomarkers as indicators of AD neuropathologic changes, integrating CSF metabolomics and genetic risk factors (i.e., polygenic risk scores, PRS) to uncover metabolic pathways involved in AD etiology and pathophysiology. For this purpose, we collected 5543 CSF mass spectrometry measurements from AD dementia patients, mild cognitive impairment (MCI) patients, and cognitively unimpaired subjects recruited from Alzheimer Amsterdam-based cohorts,^35–37^ along with genome-wide genotype data. Through correlation and elastic net regression analyses, we examined which CSF metabolites are associated with P-Tau, T-Tau, and Aβ CSF levels. Additionally, following an assessment of the contribution of *APOE* to AD PRS, we employed linear models to gauge the predictive capabilities of AD PRS for P-Tau, T-Tau, and Aβ CSF levels, and CSF metabolites. This study expands on past CSF metabolomics studies through increases in both sample size and panel of CSF metabolites measurements, identifying novel metabolites associations with P-Tau and T-Tau CSF levels that could point to metabolic pathways impacted by AD pathology.

## Methods

### Sample information and processing

#### Study participants

A total of 977 study samples (with an average age of 52.7 ± 16.6 years and 35.9% female) were used in this analysis, drawn from both a memory clinic cohort and a group of cognitively healthy subjects in the Netherlands. The memory clinic cohort was used throughout all the analysis presented in the paper, whereas the cognitively healthy cohort was only used for the polygenic risk score analysis.

The memory clinic cohort comprised samples from three cohorts affiliated with the Alzheimer Center Amsterdam. These cohorts include the Amsterdam Dementia Cohort (ADC),^35^ the 90+ Study,^36^ and the Twin Study.^37^ The ADC, initiated in the year 2000, is an ongoing observational follow-up study of patients who visited the memory clinic at Amsterdam UMC, location VU University Medical Center (VUmc), with dementia diagnoses made according to established guidelines for neurodegenerative diseases.^38–41^ The 90+ Study, part of the Innovative Medicine Initiative European Information Framework for AD (EMIF-AD), focuses on cognitively healthy individuals aged 90 and above, aiming to identify factors associated with resilience to cognitive impairment in the old.^36^ For the Twin Study, monozygotic twins (one subject per twin pair) were recruited from the Netherlands Twin Register^42^ to participate in the PreclinAD study, a component of the EMIF-AD project (http://www.emif.eu/).37 Exclusion criteria for these cohorts included the use of high-dose antidepressants, epilepsy requiring anti-epileptic drugs, and a diagnosis of schizophrenia or bipolar disorder. Cognitive status for all participants was assessed using the Mini-Mental State Examination (MMSE), an 11-item questionnaire that evaluates various cognitive domains, with scores ranging from 0 (severe impairment) to 30 (no impairment).^43^

#### Cohort of cognitively healthy subjects

The recruitment of these neurotypical subjects is described before.^23^ In short, inclusion involved patients undergoing spinal anesthesia for minor elective surgical procedures, ages between 18 and 60, and with all four grandparents born in The Netherlands or other Northwestern European countries (Belgium, Germany, UK, France, and Denmark). Each potential participant underwent a telephone interview to screen for self-reported psychotic or major neurological disorders (such as stroke, brain tumors, or neurodegenerative diseases) and to document any use of psychotropic medication.

An overview of the characteristics for the memory clinic cohort (N = 487) and the cognitively healthy subjects cohort (N = 449, all cognitive healthy controls) can be found in Table 1 and Supplemental Table 1. The ADC samples comprises n = 220 cognitively unimpaired subjects, n = 87 subjects with MCI, and n = 180 patients with AD-type dementia. As expected, patient groups in the ADC differed from each other, with the AD-type dementia group having more *APOE* ε4 carriers, fewer *APOE* ε2 carriers, and exhibiting more abnormal AD CSF biomarkers compared to the MCI and cognitively unimpaired subjects (Supplemental Figure 1). Cognitively healthy subjects, in comparison to memory clinic subjects, were less frequently female, younger, had a lower *APOE* ε4 frequency, and a higher *APOE* ε2 frequency.

**Table 1.** Cohort characteristics.

Characteristic	Clinical Cohort (n = 610)	Samples with CSF and Genotype (n = 487)
Age, mean (SD)	65.5 (9.4)	65.5 (9.7)
Female, n (%)	272 (44.6)	207 (42.5)
**Diagnosis**		
**Controls, n (%)**	**297 (48.7)**	**220** (**45.2)**
Age, mean (SD)	-	65 (12.0)
MMSE, mean (SD)	-	28 (1.4)
**MCI, n (%)**	**103 (16.9)**	**87** (**17.9)**
Age, mean (SD)	-	67 (7.7)
MMSE, mean (SD)	-	26 (2.0)
**Dementia, n (%)**	**210 (34.4)**	**180** (**36.9)**
Age, mean (SD)	-	65 (7.2)
MMSE, mean (SD)	-	21 (4.2)
***APOE* genotype**		
*ɛ*2/*ɛ*2, n (%)	-	-
*ɛ*2/*ɛ*3, n (%)	-	29 (6.0)
*ɛ*2/*ɛ*4, n (%)	-	8 (1.6)
*ɛ*3/*ɛ*3, n (%)	-	206 (42.3)
*ɛ*3/*ɛ*4, n (%)	-	170 (34.9)
*ɛ*4/*ɛ*4, n (%)	-	74 (15.2)
CSF Aβ42 (pg/ml), n (%)	-	399 (81.9)
CSF P-Tau (pg/ml), n (%)	-	407 (83.6)
CSF T-Tau (pg/ml), n (%)	-	407 (83.6)

Controls include both individuals with normal cognition and subjective cognitive decline. MCI: mild cognitive impairment; P-Tau: phosphorylated tau; T-Tau: total tau; MMSE: Mini-Mental State Examination

All participating studies were approved by their respective Medical Ethics Committees, including approval from the METC Amsterdam UMC and UCLA Institutional Review Board (IRB#20-002064). Informed consent, either from the patient or from the legal representative, was obtained from all participants. All methods were performed in accordance with the relevant guidelines and regulations.

#### CSF data collection

Memory clinic cohort: CSF samples were acquired through lumbar puncture using a 25-gauge needle and syringe, followed by storage at −80°C. Aβ, T-Tau, and P-Tau levels were assessed as part of the diagnostic work-up, employing enzyme-linked immunosorbent assays (ELISA) (Innotest: Fujirebio, Ghent, Belgium).^35^ In the ADC cohort, CSF Aβ values were adjusted for drift over time, as detailed previously.^44^ In this cohort, the biomarker abnormality cut-offs used were CSF Aβ_42_ < 813 pg/ml, CSF t-tau > 375 pg/ml, and CSF p-tau > 52 pg/ml. *Cognitively healthy subject cohort.* Each subject provided a 6 ml CSF sample through lumbar puncture, immediately stored in fractions of 0.5 and 1 ml at −80°C, as previously described.^23^

#### CSF metabolite processing

As previously described,^25^ a total of 5543 CSF metabolites were assessed for this study. CSF extraction followed a previously published protocol,^45^ using a mixture of acetonitrile/water/isopropanol (2:2:3) to precipitate proteins and extract metabolites. CSF metabolites were measured using gas chromatography (GC) mass spectrometry (MS) and two methods of liquid chromatography (LC) tandem mass spectrometry (MS/MS) at the West Coast Metabolomics Center, UC Davis. These methodologies captured different aspects of the metabolome, including GC-time-of-flight (GC-TOF) MS for primary metabolism (393 metabolites), LC charged surface hybrid quadrupole time-of-flight (CSH-QTOF) MS/MS for complex lipids (3532 metabolites), and hydrophilic interaction liquid chromatography quadrupole time-of-flight (HILIC-QTOF) MS/MS for biogenic amines (1618 metabolites). For GC-TOF MS, the 7890A Agilent™ instrument (column: Restek Rtx-5Sil MS) was used, with fragments analyzed by the Leco Pegasus IV (TOF) to obtain accurate mass information at high resolution.^46^ For MS/MS (i.e., CSH-QTOF and HILIC-QTOF), the Agilent 6530a™ was used for positive electrospray ionization (ESI) and the Agilent 6550™ for negative ESI.

Raw data processing was performed using Mass Spectrometry-Data Independent Analysis (MS-DIAL) version 4.6 and mass spectral feature list optimizer (MS-FLO).^47,48^ The data were provided as peak heights for the quantification ion (m/z value) at the specific retention time (rt value). Peak heights were used instead of peak areas because they offer greater precision for low-abundant metabolites, as baseline determinations have a larger influence on peak areas. To ensure reliable signal detection, measurements with intensities below a 10:1 ratio compared to blank controls were excluded. All metabolite intensities were normalized using Systematic Error Removal using Random Forest (SERRF), which employs pooled aliquots to normalize metabolite intensity.^49^ Each compound was identified by a unique identifier consisting of retention time (rt) and mass-to-charge ratio (m/z), annotated using the West Coast Metabolomics Center's in-house m/z-rt databases (LipidBlast spectral library for CSF-QTOF) and MS/MS spectral matching to libraries from the National Institute of Standards and Technology (NIST)^50^ and the MassBank of North America (MoNA). In total, we measured 5543 CSF compounds, including 814 annotated metabolites and 4729 unannotated compounds (Supplemental Excel file Metabolomics_meta_data includes complete mass spectrum characteristics for each metabolite, retention time, mass-to-charge ratio [m/z] and Metabolomics Standards Initiative (MSI) levels for the annotations).

To address missingness, metabolite levels were initially examined across each cohort, and metabolites with missing data for over 20% of individuals were excluded, removing 282 compounds. For the remaining 5261 CSF compounds, missing values were imputed to half the minimum value for the corresponding metabolite across the cohorts. This decision was based on the assumption that these metabolites likely exist in quantities too low to be detected in these individuals, a method consistent with previous approaches.^17^ Subsequently, inverse-rank normalization was applied to all metabolites to ensure normality for downstream analysis.

#### Genotyping and imputation

Memory clinic samples underwent genotyping using the Illumina Global Screening Array (GSA), while genotype data for cognitively healthy controls were obtained through the OmniExpress Exome array. Initial filtering of autosomal genotypes involved excluding SNPs with <2% SNP-missingness and >5% minor allele frequency (MAF) using plink,^51^ performed separately per cohort. Individuals with a call rate below 98% were excluded. Following this, genotype VCF files were uploaded to the TopMed server for imputation and liftover to hg38. Post-imputation quality was assessed by filtering variants with imputation R2 > 0.3, resulting in approximately 8 million SNPs per cohort for subsequent analyses. For the polygenic risk score analysis, imputed genotypes were subsequently merged between the two cohorts, and once again filtered for variants with <2% SNP-missingness and >5% MAF.

After correcting for ancestry PCs and age outliers (individuals that were over 3 standard deviations away from the average of the first ancestry PC, and over 100 years of age), 26 samples were removed from the memory clinic cohort, with 461 individuals remaining for the statistical analysis.

### Statistical analyses

#### Correlations between AD biomarkers and CSF metabolites

Using the residuals of linear models that included diagnosis, age and sex, individual metabolites were tested for Spearman correlation with CSF phosphorylated tau, total tau, and Aβ_1__−42_ using the cor.test function in R. Any correlations that passed Bonferroni multiple testing correction were considered significant correlations.

#### Elastic net regression

In order to restrict the number of metabolites to those most relevant to predicting AD biomarkers, elastic net regression was utilized using the train function from the caret package in R.^52^ Due to the lack of AD biomarker data in the cognitively healthy cohort, this analysis was limited to the Amsterdam based cohort. This method was implemented to select the most important metabolites out of the 5261 metabolites for predicting CSF phosphorylated tau, total tau, and Aβ_1__−42_. In the elastic net model, lambda and alpha were selected via 10-fold cross-validation. To account for variability in predictions due to differences in data partitioning, the dataset was randomly split into training (80%) and testing (20%) sets across 1000 iterations. In each iteration, a model was trained using 80% of the data to predict AD biomarkers, while the remaining 20% was used to assess prediction accuracy. To determine the added predictive value of CSF metabolites, baseline models were tested using only age and sex. Metabolites that were included in over 80% of the 1000 models were considered highly predictive. Since this approach aggregates results from multiple iterations, individual best-performing lambda and alpha values for each model are not reported. Additionally, to verify the importance of these metabolites, the rest of the metabolites that were not considered highly predictive were corrected for the effects of the highly predictive metabolites.

#### Pathway enrichment analysis

Metabolite Set Enrichment Analysis (MSEA) and PaIRKAT^53^ were employed to elucidate the biological pathways represented in metabolites of interest. MSEA through the “Pathway Enrichment” tool on MetaboAnalyst^54^ was performed on metabolites correlated with either total tau or phosphorylated tau. MSEA identified pathways whose metabolites appeared more often than chance among metabolites that correlated with tau. PaIRKAT integrates known biological connections between metabolites in pathways into a semi-parametric kernel machine regression model to associate entire pathways with phenotypes. All named metabolites that were found in the KEGG database from the three platforms were included to create networks of metabolites representing pathways. These pathways were tested for association with both phosphorylated tau and total tau while corrected for sex and age on https://csevern.shinyapps.io/pairkat/.

#### Polygenic risk scores calculation

Polygenic risk scores for AD were calculated using PRScs software^55^ and the latest AD GWAS that did not contain the cohort used in this study.^56^ We used the European reference panel from 1000 Genomes Phase 3 to model LD in the polygenic risk score computation. To improve prediction accuracy of the AD PRS, we followed the approach suggested by Leonenko et al.,^33^ in which the *APOE* locus (Chr19 43.4M-47.5 M) is removed prior to the calculation of the AD PRS, and then the *APOE* effect is added as a weighted sum to the AD PRS, with effect sizes of −0.47 for the ε2 allele and 1.12 for the ε4 allele. The region for the *APOE* locus was chosen after comparing multiple genetic windows and the resulting AD PRS across individuals with different *APOE* ε4 allele counts.

Polygenic scores for other brain disorders and traits such as schizophrenia,^57^ attention-deficit/hyperactivity disorder,^58^ bipolar disorder,^59^ insomnia,^60^ migraine,^61^ and alcohol use disorder^62^ were also calculated in both cohorts using PRScs and the respective GWAS summary statistics.

#### Association of AD PRS with CSF metabolites and AD biomarkers

Linear regression was used to identify CSF metabolites that could be predicted with the AD PRS. Using the lm function from R we generated linear models predicting CSF metabolite levels using the weighted AD PRS and as cofactors age and sex. We also compared these models to using the AD PRS calculated without the *APOE* regions, as well as including the *APOE* region. This approach was extended to associate Aβ_1__−42_, phosphorylated tau and total tau levels with the AD PRS when available for the sample. AD biomarkers levels were log normalized for linear regression analyses.

## Results

### Metabolome-wide correlation of AD biomarkers

**Figure 1.**
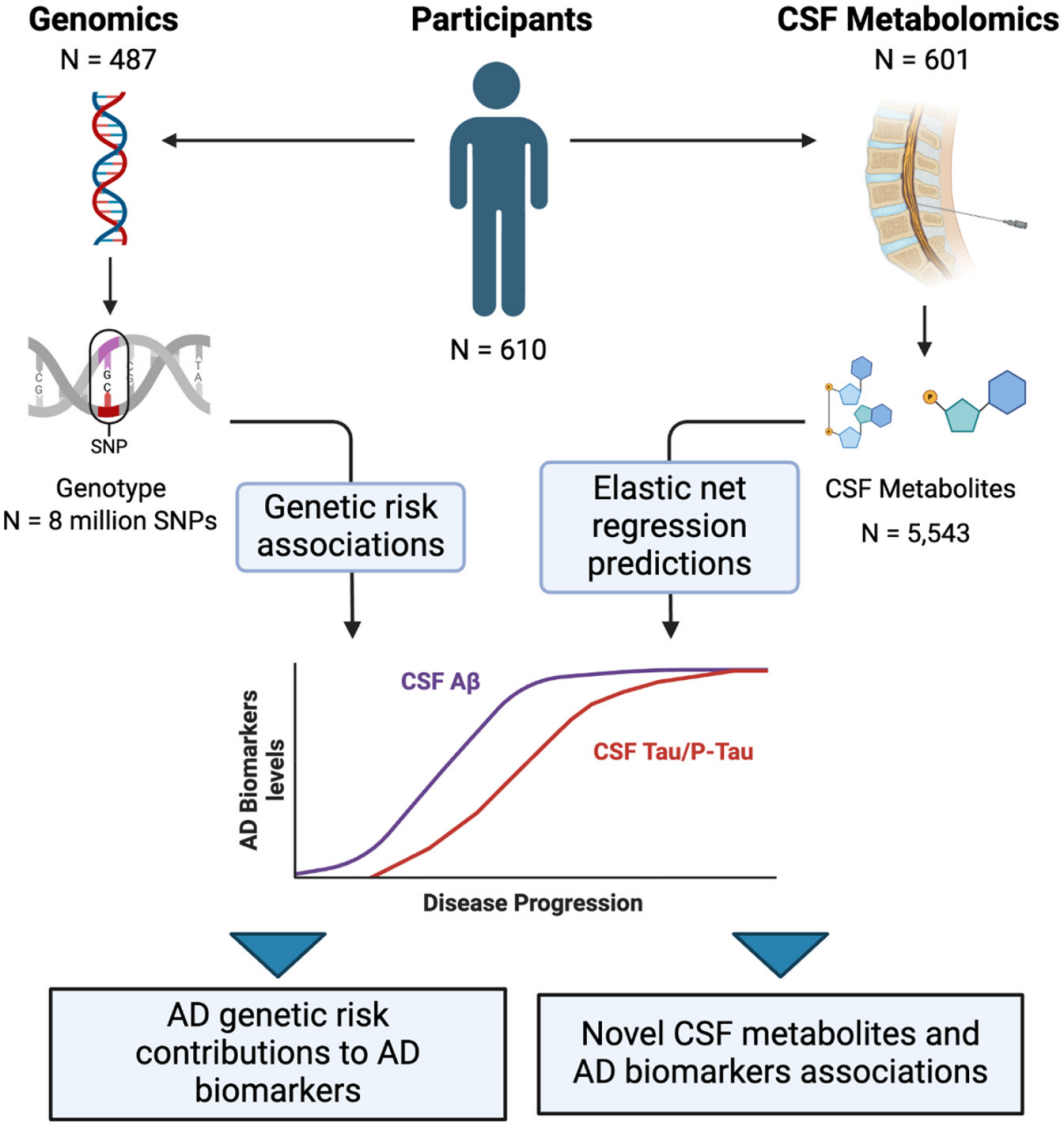
Study design outline. Single nucleotide polymorphisms (SNPs), Alzheimer's disease (AD), cerebrospinal Fluid (CSF), amyloid-β_1−42_ (Aβ), phosphorylated tau (P-Tau).

**Figure 2.**
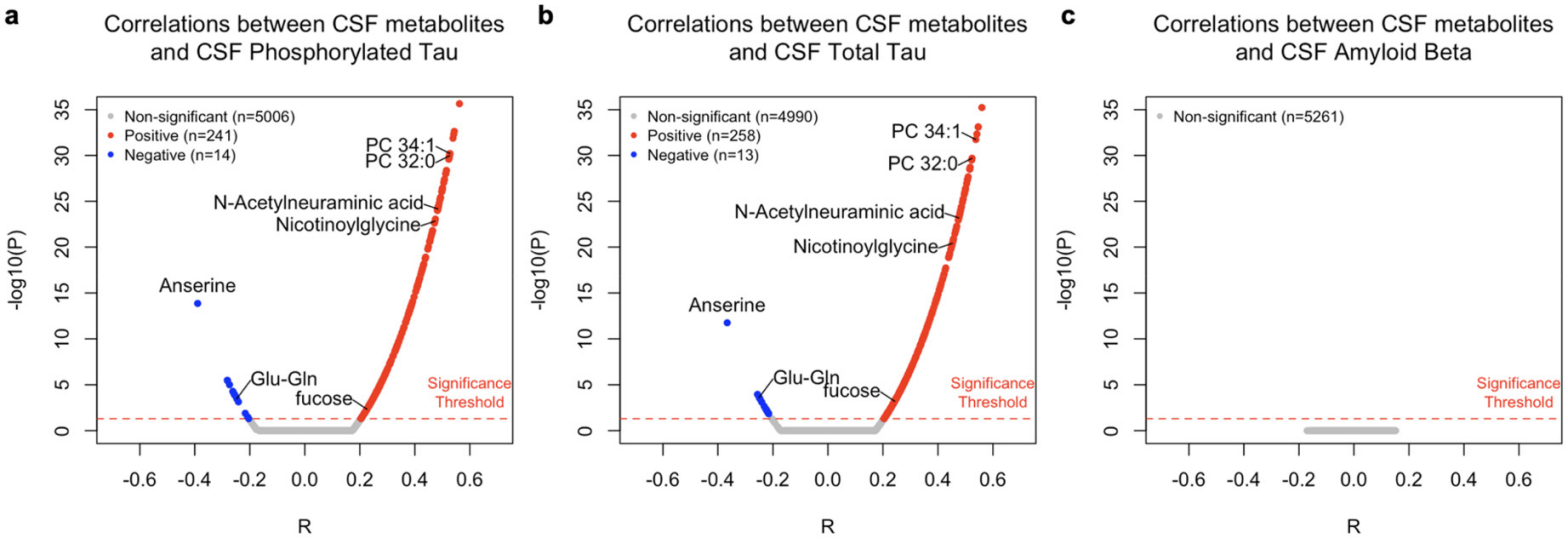
CSF metabolites correlations across CSF AD biomarkers. Correlation results between 5261 CSF compounds and CSF AD biomarkers after correcting for age and sex using the residuals from linear models. (**a**) shows results for CSF Total Tau, (**b**) for CSF Phosphorylated Tau, and **c** for CSF Aβ. X-axis represents correlation values as an adjusted R. Y-axis represents log transformed p-values. Dots indicate positive (red) and negative (blue) significantly correlated metabolites. Dotted line represents the Bonferroni adjusted significance threshold of -log10(0.05).

Abnormal levels of AD CSF biomarkers are indicative of broad AD neuropathologic changes.^63^ To assess how AD pathology influences physiological processes *in vivo*, we examined the relationships between CSF metabolites and CSF levels of P-Tau, T-Tau, and Aβ. The study design is outlined in Figure 1. CSF samples were collected and processed from 601 individuals recruited from three previously established Alzheimer Amsterdam-based cohorts (see Methods section for descriptions of these cohorts).^35–37^ Participants included individuals with normal cognition (NC), subjective cognitive decline (SCD), MCI, and AD. Table 1 summarizes the characteristics of the cohort. Untargeted metabolomic data were collected from three distinct platforms capturing metabolites across primary metabolism,^64^ complex lipids,^65^ and biogenic amines^66^ (see Methods section for full details). After removing 282 compounds due to missingness, we analyzed the remaining 5261 CSF compounds (678 named metabolites, 4583 unnamed compounds). Of these, 271 (120 named metabolites) demonstrated significant correlations with T-Tau CSF levels after correcting for diagnosis, age, and sex, with Bonferroni correction for multiple testing (Figure 2(a)). Among them, 258 exhibited positive correlations, while 13 showed negative correlations. Similarly, 255 compounds (108 named metabolites) were correlated with P-Tau, with 241 showing positive correlations and 14 showing negative correlations (Figure 2(b)). Table 2 summarizes the strongest positive and negative correlations of named CSF metabolites for both T-Tau and P-Tau.

**Table 2.** Summary of the strongest CSF metabolite correlations for T-Tau and P-Tau CSF levels.

Metabolites Correlations				
	CSF Phosphorylated Tau		CSF Total Tau	
CSF Metabolite	R	Adjusted p	R	Adjusted p
PC 34:0	0.539	1.28E-32	0.523	3.16E-30
PC 34:1	0.528	5.99E-31	0.538	1.80E-32
PC 32:0	0.526	1.07E-30	0.524	2.01E-30
PC 40:4	0.493	4.30E-26	0.477	3.95E-24
PI 40:6	0.486	3.43E-25	0.462	3.44E-22
PE 36:1	0.486	3.52E-25	0.510	2.11E-28
N-Acetylneuraminic acid	0.483	6.81E-25	0.476	6.40E-24
PC 36:1	0.482	1.01E-25	0.482	9.32E-25
Nicotinoylglycine	0.473	1.48E-23	0.453	3.91E-21
PI 38:5	0.471	2.2E-23	0.467	8.29E-23
Glu-Gln	-0.247	3.38E-04	-0.250	2.40E-04
Anserine	-0.390	1.39E-12	-0.366	1.78E-12

Summary of the strongest named CSF metabolite correlations with Total Tau and Phosphorylated Tau CSF levels. The first ten rows represent significant positive correlations, while the last two rows represent significant negative correlations. A black line separates the positive and negative correlations for clarity.

A total of 242 compounds (98 named metabolites) were correlated with both P-Tau and T-Tau CSF levels, all in the same direction (Supplemental Figure 2). 13 compounds were uniquely associated with P-Tau, while 29 were uniquely associated with T-Tau (full list available in the Supplemental Material). Notably, no metabolites or compounds were significantly correlated with CSF Aβ levels (Figure 2(c)). To determine whether these associations were specific to AD, we stratified the cohort by cognitive status and repeated the correlation analysis. The relationships between CSF metabolites and CSF T-Tau/P-Tau levels remained largely consistent between cases (MCI and AD) and controls (NC and SCD) (Supplemental Figure 3). No significant differences were observed in correlation coefficients across groups for any measured CSF metabolites. Furthermore, when performing the correlation analysis without accounting for diagnosis, 208 of the significant CSF compounds associated with T-Tau and 214 with P-Tau were replicated. This suggests that most metabolite associations with CSF T-Tau and P-Tau levels are independent of AD case-control status in this cohort.

### CSF metabolites can predict AD biomarkers

To further refine our findings and identify combinations of CSF compounds that could jointly predict P-Tau and T-Tau CSF levels, we employed elastic net regression models. These models utilized our CSF compounds measurements as predictors, alongside age and sex, for P-Tau and T-Tau CSF levels. To identify consistently predictive CSF compounds, we constructed these elastic net regression models over a thousand iterations of training and testing sets. In each iteration, 80% of the samples from our cohort were randomly selected for the training set, while the remaining 20% were used as the test set. Metabolites present in 80% of these models were considered consistent predictors. Base models using only age and sex as predictors were also constructed for comparison.

**Figure 3.**
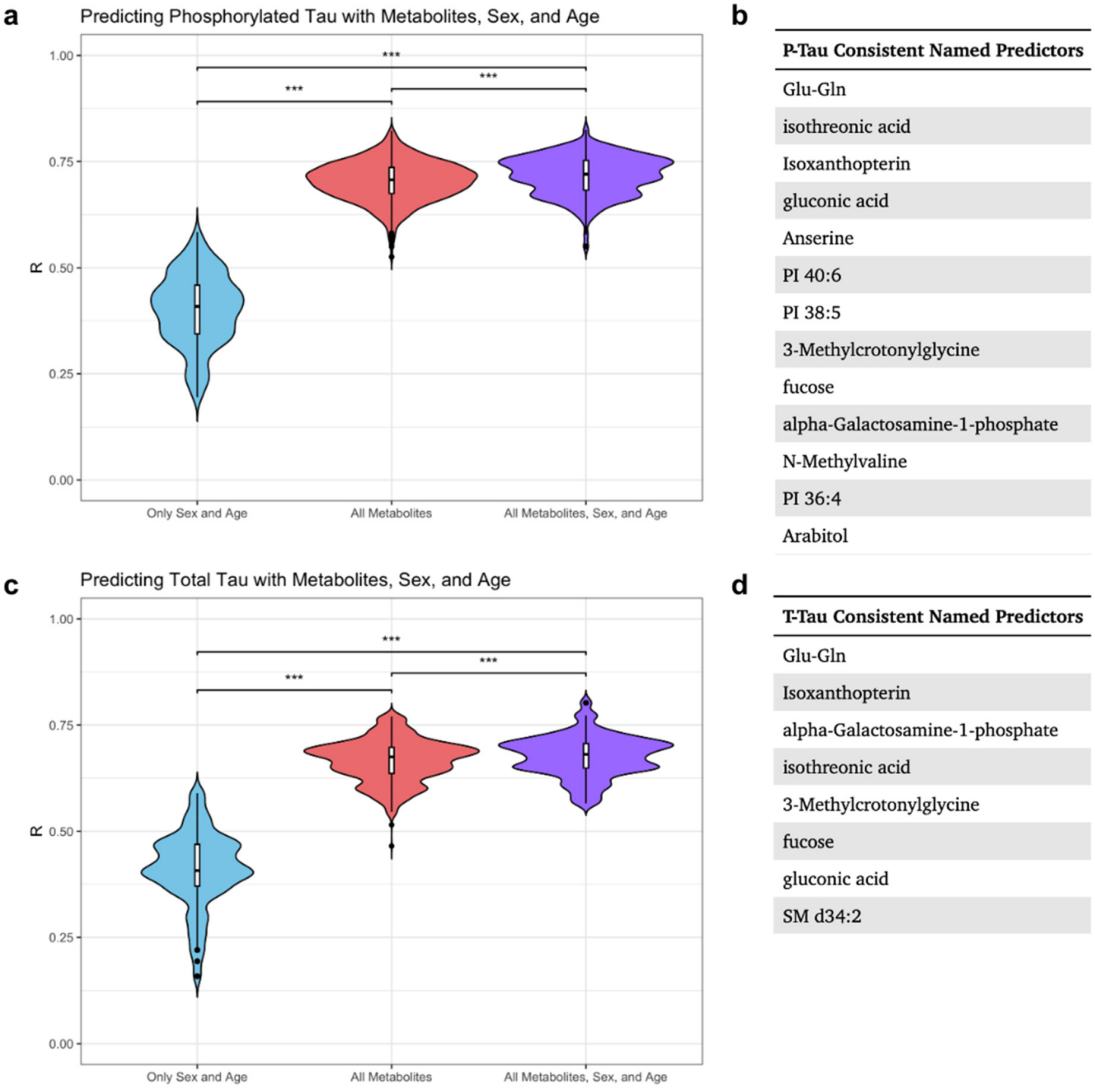
Prediction of P-Tau and T-Tau CSF levels using CSF metabolites. Elastic net prediction results of P-Tau (**a**) and T-Tau (**c**) CSF levels using only age and sex, all the CSF compounds (n = 5261), and a combined model of both. Y-axis represents R values for the models. (**b**) and (**d**) show all named CSF metabolites that consistently came up as predictors for P-Tau (n = 13) and T-Tau (n = 8) respectively. ***p-value below 0.0001.

Models using only age and sex as predictors had an average R of 0.402 for P-Tau (Figure 3(a)) and 0.410 for T-Tau (Figure 3(c)) across the 1000 iterations. Upon adjusting for age and sex, elastic net regression models incorporating CSF compounds demonstrated an average R of 0.717 for P-Tau (Figure 3(a)) and 0.677 for T-Tau (Figure 3(c)), a significant increase compared to the average from the base model (p < 2e-16 for both P-Tau and T-Tau). For models predicting P-Tau CSF levels, 41 compounds were identified as consistent predictors, with 13 being named metabolites (Figure 3(b)). Of these, 7 were identified on the biogenic amines platform (Glu-Gln, Isoxanthopterin, Anserine, Arabitol, N-Methylvaline, 3-Methylcrotonylglycine, Alpha-Galactosamine-1-phosphate), 3 from the complex lipids platform (PI 40:6, PI 38:5, PI 36:4), and 3 from the primary metabolism platform (Isothreonic acid, Gluconic acid, Fucose). For models predicting T-Tau CSF levels, 33 compounds were identified as consistent predictors, with 8 being named metabolites (Figure 3(d)) (full list provided in the Supplemental Material). Except for the SM d34:2 metabolite, a phosphosphingolipid, all consistent named metabolites predicting T-Tau CSF levels were also highly predictive of P-Tau CSF levels. We also applied this approach to Aβ CSF levels to assess if this method could identify CSF metabolites predictive of Aβ CSF levels. Although including these CSF compounds in the elastic net models provided additional predictive power for P-Tau and T-Tau CSF levels, they did not significantly increase the average prediction accuracy of Aβ CSF levels compared to the base model using only age and sex (Supplemental Figure 4). Due to the limitations of including unknown CSF compounds, we repeated this analysis using only identified CSF metabolites (n = 678). Models based solely on this subset showed lower average predictive power compared to those using all available measurements (n = 5261) (Supplemental Figure 5), which is expected given the smaller number of variables. However, this approach successfully replicated the same set of metabolites for T-Tau CSF levels and nearly all for P-Tau levels, with the exception of PI 36:4. A summary of these results are provided in the Supplemental Material.

### Pathway analysis of CSF metabolites correlated with P-tau and T-tau

**Figure 4.**
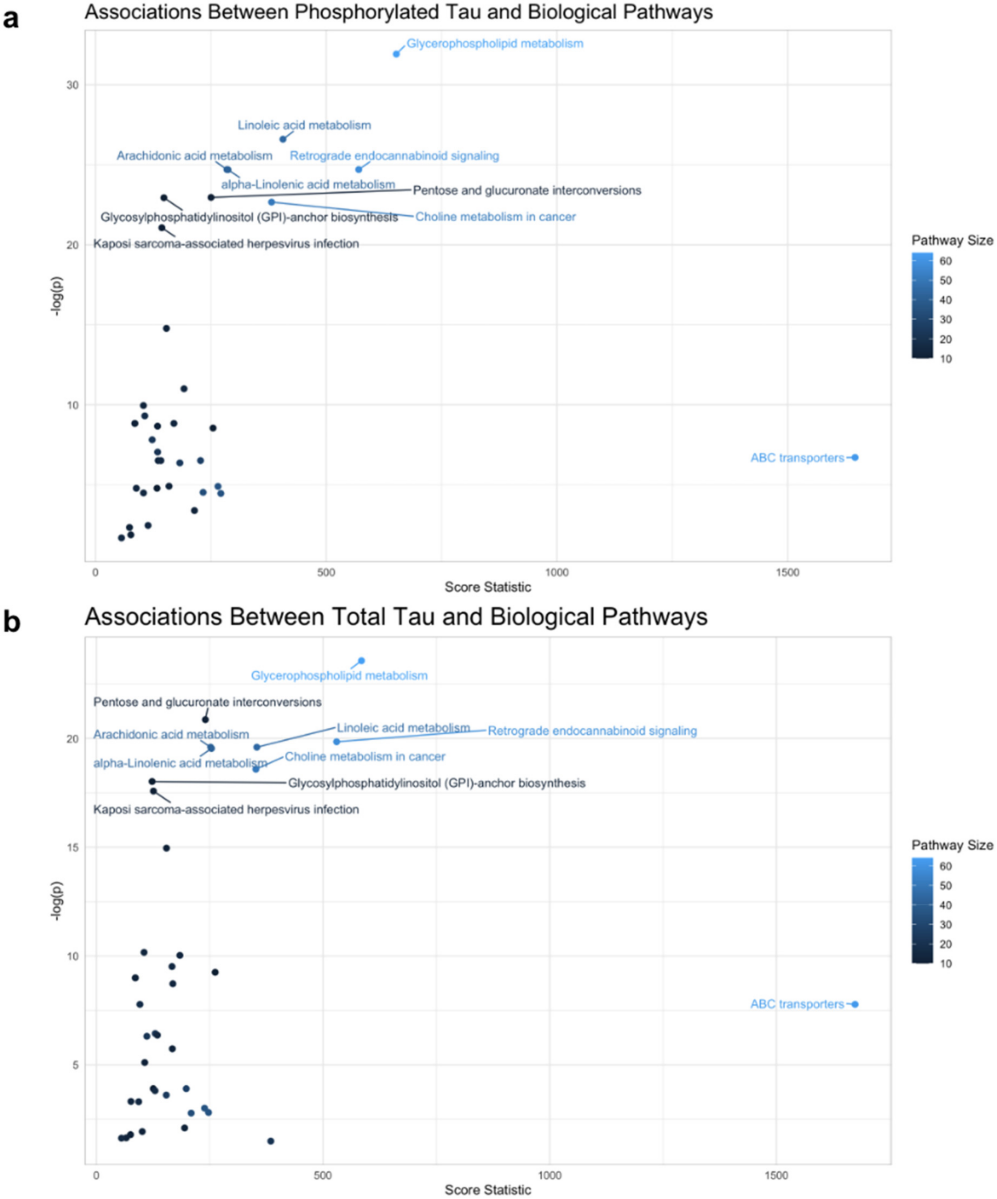
Pathway analysis of metabolites correlated with P-Tau and T-Tau. Results of PaIRKAT pathway enrichment analysis. All 678 named CSF metabolites were included to create networks of metabolites representing pathways that were tested for association with either P-Tau (**a**), or T-Tau CSF levels (**b**). X-axis represents the kernel association test enrichment score; Y-axis represents log transformed p-values. Color indicates the number of metabolites that were included for that particular pathway.

Having identified metabolites and compounds that are correlated with, and provided consistent predictions for P-Tau and T-Tau CSF levels, our next step was to test which metabolic pathways are associated with these metabolites. Based on the 242 unique compounds that correlated with either P-Tau or T-Tau, we conducted an overrepresentation analysis (see methods section for details). This analysis revealed three significantly enriched pathways: the Pentose and Glucuronate Interconversions, Ascorbate and Aldarate Metabolism, and Amino Sugar and Nucleotide Sugar Metabolism (Supplemental Figure 6). To corroborate these findings, we employed PaIRKAT,^53^ a pathway-integrated regression-based kernel association test that associates entire pathways with a phenotype. This analysis reaffirmed our results, identifying the Pentose and Glucuronate Interconversions pathway, as well as additional pathways that were significantly associated with both P-Tau (Figure 4(a)) and T-Tau (Figure 4(b)) levels. Many of the highly enriched pathways overlapped between the P-Tau and T-Tau results, including Glycerophospholipid Metabolism, ABC Transporters, Linoleic Acid and Arachidonic Acid Metabolism, Retrograde Endocannabinoid Signaling, Alpha-Linoleic Acid Metabolism, and Choline Metabolism in Cancer (full list of enriched metabolites in the Supplemental Material).

### Prediction of AD biomarkers and CSF metabolites using polygenic risk scores

Previously, it has been shown that AD biomarker levels in CSF (i.e., P-Tau, T-Tau, and Aβ) are associated with the polygenic risk of AD.^3^ To investigate similar associations in our clinical cohort, we constructed a PRS for AD based on the most recent GWAS that did not include our study cohort.^56^ Due to the significant impact of the *APOE* locus on the PRS (Supplemental Figure 7), we used an approach that initially generates the AD PRS without the *APOE* locus and subsequently incorporates the effects of the *APOE* ε4 and ε2 alleles as a weighted sum (*APOE-*weighted PRS).^33^ After adjusting for age and sex, linear models revealed that the *APOE*-weighted AD PRS was significantly correlated with Aβ CSF levels (Adjusted R^2^ = 0.220; p = 9.11E-14) but yielded only a modest association signal with P-Tau (Adjusted R^2^ = 0.137; p = 0.014) and T-Tau (Adjusted R^2^ = 0.144; p = 0.007) CSF levels.

We then compared various models, incorporating different approaches to account for the effects of the *APOE* alleles. These approaches included using (i) only the *APOE* ε4 allele counts, (ii) the AD PRS without the *APOE* region, and (iii) a weighted risk score for *APOE* based on autopsy-confirmed AD cases (*APOE*-npScore).^67^ The summarized results (Table 3) revealed that across all three biomarkers, the AD PRS without the *APOE* region explained the least variance (Aβ R^2^ = 0.102, P-Tau R^2^ = 0.129, and T-Tau R^2^ = 0.134). Additionally, the AIC (Akaike information criterion) and BIC (Bayesian information criterion) model selection criteria supported the *APOE*-weighted AD PRS over all other tested models (Aβ AIC = 186.94, BIC = 206.7; P-Tau AIC = 554.45, BIC = 573.4; and T-Tau AIC = 771.05, BIC = 790.9). However, its improvement for the variance explained over solely using the *APOE* ε4 allele counts was marginal for P-Tau (R^2^ = 0.137 versus 0.135) and T-Tau (R^2^ = 0.144 versus 0.142), and equal for Aβ (R^2^ = 0.220 versus 0.220).

**Table 3.** AD biomarkers prediction results.

	CSF Amyloid-Beta						CSF Phosphorylated Tau						CSF Total Tau					
Model	n	R2	AIC	BIC	Beta	p	n	R2	AIC	BIC	Beta	p	n	R2	AIC	BIC	Beta	p
**ε4 allele counts**	394	0.220	187.04	206.8	−0.346	9.59E-14	402	0.135	555.23	575.1	0.108	0.021	402	0.142	771.94	791.8	0.119	0.011
***APOE* weighted AD PRS**	394	0.220	186.94	206.7	-0.347	9.11E-14	402	0.137	554.45	574.3	0.116	0.014	402	0.144	771.05	790.9	0.126	0.007
***APOE*-npScore**	394	0.217	188.42	208.1	-0.343	1.91E-13	402	0.136	555.09	574.9	0.110	0.0197	402	0.142	771.72	791.5	0.121	9.97E-03
**AD PRS without *APOE* region**	394	0.102	242.55	262.3	-0.036	0.4500	402	0.129	557.89	577.7	0.076	0.1023	402	0.134	775.62	795.46	0.078	0.0955

Summary of results from linear regression models, after adjusting for age and sex, using different approaches to account for the *APOE* ε4 allele and genetic architecture of AD. R2: R-squared; AIC: Akaike information criterion; BIC: Bayesian information criterion.

While metabolomics can offer dynamic insights into an individual's physiological state and environmental exposures, multiple CSF metabolites are under genetic control.^23,24^ To investigate metabolic processes associated with the genetic architecture of AD, we applied linear models to predict the levels of 5261 CSF compounds using the *APOE*-weighted AD PRS, adjusting for age and sex. This analysis identified four significant associations (FDR < 0.05) between phosphatidylcholines (PC) and the *APOE*-weighted AD PRS: PC 30:0 (R² = 0.130, p = 8.20e-07), PC 32:0 (R² = 0.135, p = 3.56e-07), PC 32:0 (5.60_792.58) (R² = 0.107, p = 7.30e-07), and PC 34:0 (R² = 0.123, p = 1.30e-09). A follow-up analysis using *APOE* ε4 allele counts instead of the *APOE*-weighted AD PRS yielded similar results, suggesting that these associations were primarily driven by the *APOE* ε4 allele (Supplemental Figure 8 and the Supplemental Material). To validate these results and assess whether these associations between the *APOE*-weighted AD PRS and CSF metabolites are specific to a cohort with an AD genetic background, we replicated our analysis on an unrelated, cognitively healthy cohort (n = 449) with the same panel of CSF compounds measurements.^23^ These participants underwent spinal anesthesia for minor elective surgical procedures and were screened for self-reported psychotic or major neurological disorders (Further cohort description in the Methods section and Supplemental Table 1). Linear modeling in this cohort failed to reveal any significant associations between CSF metabolites and the *APOE*-weighted AD PRS, both nominally and after multiple testing corrections (Supplemental Figure 8 and the Supplemental Material). To investigate if the absence of associations was exclusive to the AD PRS, we expanded our analysis to include other brain disorders and traits such as schizophrenia,^57^ attention-deficit/hyperactivity disorder (ADHD),^58^ bipolar disorder,^59^ Insomnia,^60^ migraine,^61^ and alcohol use disorder.^62^ A summary of these associations can be viewed in Supplemental Figure 8. None of these polygenic scores yielded significant associations with CSF metabolites in either the clinical cohort or the cognitively healthy cohort.

The absence of associations may also be influenced by the age difference between cohorts, as the cognitively healthy cohort was younger (mean age 39.7 ± 11.4 years) than the clinical cohort (mean age 65.5 ± 9.7 years). Given that aging is a key contributing factor to AD pathology,^68^ we repeated the analysis after stratifying the clinical cohort by age: younger individuals (< 60 years, N = 124) and older individuals (>= 60 years, N = 337). In this stratified analysis, only the association between PC 34:0 and the AD PRS remained significant in the older subgroup, while no significant associations were observed in the younger subgroup. The lack of associations in the younger subgroup may reflect reduced statistical power due to sample size, but it may also suggest that these associations are age-dependent. To further investigate whether these findings are specific to AD rather than general age-related effects, we stratified the older subgroup by AD diagnostic status into cases (N = 206) and controls (N = 131). No significant associations were identified in either group, likely due to limited statistical power. Full results of these analyses are provided in the Supplemental Material.

## Discussion

By leveraging genotype data, an extensive panel of 5261 CSF metabolites, and AD CSF biomarkers in a large cohort, in this study we aimed to elucidate biological pathways impacted by the neuropathologic changes associated with AD. Our correlation and elastic net regression analyses revealed 242 unique CSF compounds linked with P-Tau and T-Tau CSF levels, yet no metabolites demonstrated associations with Aβ CSF levels. Among the CSF metabolites predictive of both P-Tau and T-Tau levels found in this study, we identified Anserine and Fucose as novel associations. The pathway enrichment analysis of these CSF metabolites associated with P-Tau and T-Tau levels consistently highlighted pentose and glucuronate interconversions, as well as glycerophospholipid metabolism as significant pathways for both AD biomarkers. Furthermore, we have also identified 3 unique CSF metabolites associated with PRS of AD adjusted for the substantial effect size of *APOE* alleles, all of which were phosphatidylcholines. Linear regression models did not replicate these associations between CSF metabolites levels and the *APOE*-weighted AD PRS in our cognitively healthy cohort, nor further associations were identified for additional polygenic scores related to various brain traits and disorders. With this AD PRS, we also observed a significant prediction for Aβ CSF levels but with only limited and modest predictive value to either P-Tau and T-Tau CSF levels. Notably, the AD PRS without the *APOE* locus yielded the lowest-performing predictions across all three biomarkers, thereby reiterating the known importance of the *APOE* locus as an AD risk factor.

After assessing the strength and directionality of the associations between CSF compounds and AD CSF biomarkers, we identified 204 CSF compounds that significantly correlated with P-Tau CSF levels and also with T-Tau CSF levels, reflecting the high correlation between these two biomarkers.^69^ This number represents a significant increase compared to previous studies, which identified 38 CSF metabolites associated with both P-Tau and T-Tau CSF levels.^15,17^ While these prior studies investigated CSF metabolites in relation to AD biomarkers, differences in metabolite panels present a challenge for direct comparisons across cohorts. Nonetheless, we independently replicated 14 of these previously reported associations and confirmed that metabolites such as Cholesterol, N-acetylserine, and Arabitol are associated with these AD biomarkers (see the Spplemental Material for the full comparison). Previous metabolomic studies on plasma have also identified associations between CSF P-Tau, T-tau and metabolites, such as glutamine,^70^ kynurenine and picolinic acid,^71^ and sphingolipids.^72^ However, the presence of the blood-brain barrier creates a challenge for direct comparisons between the biofluids,^16^ as other studies have shown different degrees of correlations between metabolite levels.^13–15^ Interestingly, we found that most of the significant correlations between CSF metabolites and P-Tau/T-Tau CSF levels were independent of diagnosis in this cohort. This lack of a diagnosis effect may reflect how these biomarkers influence cognition. While CSF AD biomarkers capture the pathological changes in the brain associated with AD, their relationship with different cognitive phenotypes remains under investigation.^69,73^ A previous study highlighted that in MCI individuals, CSF levels of Aβ and P-Tau differentially predicted cognitive functions such as learning, working memory, and processing speed.^74^ As diagnosis categories primarily reflect broad cognitive impairments rather than specific cognitive dimensions, future studies assessing the relationship between CSF Tau, P-Tau, and associated metabolites with distinct cognitive functions could provide insights into biological pathways contributing to specific cognitive deficits in AD.

Among the CSF metabolite associations identified in our study using both correlation analysis and elastic net regression, we also identified two novel associations: Anserine and Fucose. Anserine is the methylated analogue of carnosine, an endogenous dipeptide present in various mammalian tissues, including the brain.^75^ Due to its antioxidant and anti-inflammatory functions, carnosine could potentially modulate biological pathways affected by AD.^76^ Supplementation with anserine and carnosine has demonstrated improvements in AD symptoms in both AD mouse models^77^ and elderly healthy human subjects,^78^ particularly in alleviating memory deficits. The observed strong negative correlation between anserine and P-Tau/T-Tau aligns with a potential protective mechanism, though further studies are essential to elucidate the interactions of anserine with these AD biomarkers. The other newly identified metabolite, Fucose, is a sugar component of glycolipids and glycoproteins.^79^ Fucose's incorporation into glycolipids has been linked to effects on learning, long-term potentiation, and synapse formation in animal models.^80,81^ Direct delivery of fucose into the brain improved retention of learned behavior in rats^82^ and mitigated neuroinflammation by inhibiting microglial cell activity in mice.^83^ While these functions require validation in humans, a transcriptomic study of brain samples from an AD cohort identified the upregulation of the FUT8 gene in AD subjects.^84^ The protein product of FUT8 catalyzes fucose transfer to glycolipids,^85^ highlighting the potential role of fucosylation in AD.

The pathway enrichment analysis of the CSF metabolites associated with P-Tau and T-Tau levels consistently highlighted glycerophospholipid metabolism as a significant pathway for both AD biomarkers. Glycerophospholipids, major components of cellular membranes, play a diverse role in altering the functional properties of cells, including signal transduction, vesicle trafficking, and membrane fluidity.^86^ In the brain, phospholipases actively catabolize glycerophospholipids through hydrolysis.^87^ Abnormal lipid metabolism, particularly changes in glycerophospholipids, has been linked to AD pathogenic features such as amyloidogenesis, oxidative stress, and neuroinflammation.^88^ A postmortem lipidomics study on brain tissue identified glycerophospholipids among the lipid subclasses significantly perturbed in AD cases.^89^ Among the metabolites identified by elastic net regression, three glycerophosphoinositols (PI 40:6, PI 38:5, PI 36:4) exhibited the highest positive correlations with P-Tau and T-Tau. These metabolites are derived from membrane phosphoinositides via the enzymatic activity of phospholipase A2 and lysophospholipase.^90^ While abnormal phospholipase A2 activity has been proposed as a predictor of cardiovascular disease, its role in dementias such as AD remains unclear.^91^ The enrichment of CSF metabolites in glycerophospholipid metabolism observed in our study underscores the relevance of this biological pathway in AD pathology. Another enriched pathway was pentose and glucuronate interconversions, previously associated with metabolites linked to P-Tau/T-Tau in an independent but much smaller AD cohort.^17^ Although dysregulation of glucose and pentose brain levels has been associated with AD pathology,^92,93^ the specific interactions of these pathways and associated metabolites with Tau in AD remain to be elucidated. The enrichment of these pathways in metabolites strongly correlated with P-Tau/T-Tau suggests a potential association between elevated levels of these AD biomarkers and processes specifically impacting neuronal activity and viability. A prior study, integrating proteomic, genomic, and imaging data, demonstrated that distinct proteomic profiles and associated pathways were linked to varying levels of P-Tau/T-Tau within individuals with AD.^94^ Further exploration of the data generated in this study offers an opportunity to assess whether different metabolites and pathways are associated with diverse levels of these AD biomarkers.

The absence of associations between CSF metabolites and Aβ levels may be attributed to the early accumulation of Aβ in the brain.^1^ Although Aβ is an established biomarker for AD, its accumulation occurs early in the disease process and is not strongly predictive of disease progression, unlike P-Tau and T-Tau CSF levels.^95,96^ These temporal characteristics suggest that the metabolic impact of Aβ may be more detectable in younger individuals, prior to the onset of AD. Future metabolomic studies in younger or preclinical cohorts could capture the early metabolic alterations related to Aβ. Consistent with previous research using smaller cohorts, our larger study also found no significant associations between CSF metabolites and Aβ levels.^15,17^ Despite significantly expanding the metabolite panel by nearly tenfold compared to prior work, it remains possible that specific Aβ metabolic signals were missed. The effects of Aβ on the CSF metabolome may be subtle, requiring even larger cohorts or more sensitive detection methods to identify. Larger, and longitudinal metabolomic cohorts that can capture disease severity progression at an individual level, as well as improvements in metabolite profiling technologies, may hold promise for elucidating the intricate relationship between Aβ accumulation and CSF metabolites.

In our analysis of the genetic architecture of AD and AD CSF biomarkers, the ε4 allele counts alone significantly predicted P-Tau, T-Tau and Aβ CSF levels, corroborating findings from previous studies.^67,97^ The *APOE* gene, encoding a glycoprotein lipid transporter, exhibits isoform-specific impacts on its function.^98^ These results align with the proposed relationship between Aβ and the *APOE* locus, where Aβ plaque levels are influenced by different *APOE* alleles.^99^ Regarding Aβ, it is hypothesized that the ε4 allele may influence Aβ levels by promoting plaque formation^100^ and hindering its clearance from the brain.^101^ *APOE* alleles have also been related to differences in Tau neurofibrillary tangles CSF levels,^102^ although the precise association between *APOE* and Tau is still under investigation. Our findings align with these known interactions and support prior reports identifying the *APOE* locus as a major genetic risk factor for Aβ, T-Tau, and P-Tau CSF levels.^3,4,32^ While this study directly compares the AD PRS with AD CSF biomarkers, alternative multi-omics approaches can provide valuable insights. For example, a previous study demonstrated the benefits of integrating metabolomics and genetic data, showing that a model combining an AD polygenic risk score with a metabolomic score derived from plasma metabolites yielded the highest hazard ratio for AD.^103^

Metabolite levels can be heritable traits, as demonstrated by prior QTL mapping studies.^20,22,25,26,104^ For instance, polygenic scores of trait measures, such as body mass index, have been associated with metabolite levels.^34^ Our analysis revealed four significant associations between CSF metabolite levels and the *APOE*-weighted AD PRS, with no additional associations observed for other polygenic scores related to brain traits or disorders. Notably, the identified metabolites were phosphatidylcholines (PC 30:0, PC 32:0, and PC 34:0), which are among the most abundant phospholipids in mammalian cell membranes and play key roles in lipoprotein transport, cell signaling, and energy metabolism.^105^ Phosphatidylcholines have also been implicated in neuroplasticity, either through direct activation of intracellular neuronal signaling pathways or by modulating membrane function.^106^ Altered phosphatidylcholine metabolism may represent a component of the broader dysregulation of lipid metabolism implicated in AD pathophysiology. Plasma lipidomics studies have reported decreased phosphatidylcholine levels in AD,^107,108^ and CSF lipidomics studies have identified significant differences in specific phosphatidylcholine species between AD and controls,^107^ including PC 32:0.^109^ Further analysis revealed that *APOE* ε4 allele counts were the primary driver for these associations. Modifications of lipid metabolism have been linked to the different isoforms of the APOE,^106^ as these are known to impact its function.^98^ For example, the *APOE* ε4 allele has been linked to cholesterol and triglyceride accumulation in glial cells.^110^ Although the direct interaction between APOE isoforms and phosphatidylcholines remains unclear, a biochemical study suggested that phosphatidylcholine clearance rates differ among APOE isoforms.^111^ While there is evidence linking phosphatidylcholines to AD and *APOE* genotype, we were unable to replicate our findings in an independent, cognitively healthy cohort. A likely explanation is the demographic differences between cohorts, as the cognitively healthy cohort was younger (mean age: 39.7 ± 11.4 vs. 65.5 ± 9.7 years), predominantly male (27.6% female vs. 42.5%), and had fewer *APOE* ε4 allele carriers (33.9% vs. 51.7%). To explore the impact of age, we stratified the clinical cohort into younger (< 60 years) and older (>= 60 years) subgroups. This analysis showed that only the association between PC 34:0 and the AD PRS remained significant in the older group, with no significant associations found in the younger group. Further stratification of the older individuals into AD cases and controls to disentangle general age-related effects from those specific to AD did not yield any significant associations, likely due to limited statistical power. Although the current dataset could not be applied to distinguish between age-related and AD specific effects, these results highlight the impact of age on our associations. In addition to age, both sex and APOE genotype are known to influence lipid metabolism, including phosphatidylcholine levels.^110,112,113^ A study of 1517 individuals further demonstrated that phosphatidylcholine serum levels are significantly associated with both sex and *APOE* genotype.^114^ Although the metabolite panels were comparable between cohorts, these demographic disparities likely contributed to the differences in findings. We encourage future studies to consider these demographic factors when attempting to replicate metabolomic associations in independent cohorts.

Our correlation analyses showed both positive and negative relationships between CSF metabolites and AD biomarkers, while the elastic net regression and pathway enrichment analyses added further evidence for their biological relevance. However, determining whether these metabolic patterns reflect downstream responses to these AD biomarkers will require comprehensive investigations into the broader pathways in which these metabolites are part of. Furthermore, while our multi-level analyses provide converging evidence supporting the relevance of these metabolites in relation to both P-Tau and T-Tau, as well as to AD pathology more broadly, our study does not directly assess the mechanistic interactions between these metabolites and AD. To establish causal links and functional relevance, future research should incorporate molecular and cellular studies aimed at dissecting how these metabolic changes are triggered or influenced by neurodegenerative processes that are part of AD. Another primary limitation of this study is the current inability to validate the strength of our predictors for P-Tau and T-Tau on an independent cohort. Although we attempted to mitigate this limitation by generating 1000 predictive models and summarizing the results, our findings still represent an overfit to this particular dataset. Furthermore, due to the lack of overlap in the panels of metabolites used, direct comparisons across cohorts used in different publications are particularly challenging. Standardizing the panels used in metabolomic studies and applying a meta-analysis approach could improve the detection of biological signals associated with AD. The metabolome encompasses chemicals from core nutrient metabolism, lipids, diet-derived compounds, and pharmaceuticals,^115^ capturing multiple exposures that influence an individual's metabolic profile. In this study, we adjusted for age and sex—two major determinants of metabolic variation—but were unable to account for polypharmacy. Polypharmacy, defined as the concurrent use of five or more medications,^116^ affects up to two-thirds of older adults worldwide.^117^ In the context of AD, it often includes cognitive-enhancing drugs (e.g., acetylcholinesterase inhibitors, NMDA receptor antagonists) alongside psychotropic medications such as antidepressants, antiepileptics, antipsychotics, opioids, and benzodiazepine receptor agonists.^118^ The effects of polypharmacy on both the metabolome and AD pathology remain poorly understood, as research has primarily been limited to animal models,^119,120^ and sex-specific consequences in humans are still under investigation.^121^ Future studies with emerging precision medicine approaches that integrate metabolomics and detailed pharmacological profiles^122^ may offer new insights into the interplay between polypharmacy and AD. Another limitation is that the cohort utilized in this study comprises exclusively individuals of European ancestry, restricting the generalizability of the results to other ancestry groups. Both the genetic architecture of AD and the performance of AD biomarkers exhibit variations across populations of different ancestries, such as in the case of African American populations. Although the number and size of GWAS studies on African American individuals with AD are limited,^123^ evidence suggests that, besides the *APOE* locus, the *ABCA7* locus shows strong associations with AD in this population.^124^ Similarly, the performance of T-Tau and P-Tau as biomarkers for AD is inconsistent in African American populations.^125^ One study demonstrated significantly lower levels of these metabolites in African Americans compared to non-Hispanic white individuals.^126^ Future AD studies should aim to broaden their cohorts to include diverse populations, enhancing our understanding of the disease across different ancestry groups.

In this study, our aim was to assess how distinct biological layers, including genetic risk and metabolomic profiles, contribute to capturing various aspects of AD pathology associated with the established clinical biomarkers of disease. We successfully identified and replicated findings regarding the impact of the *APOE* ε4 allele on Aβ, P-Tau, and T-Tau CSF levels. Additionally, we compared the effectiveness of different approaches in modeling the genetic architecture of AD. We have also extended prior CSF metabolite investigations by substantially increasing the cohort size as well as by significantly expanding the scope of the metabolomic measurements (with more than five thousand compounds). This increased effort resulted in the identification of 242 unique CSF compounds associated with CSF levels of P-Tau and T-Tau, but also highlighted the lack of any significant findings of an association between CSF metabolites and Aβ. We identified novel associations between the CSF metabolites Anserine and Fucose, and P-Tau/T-Tau. Pathway analysis of these metabolites further supports their involvement in established biological pathways affected by AD. This makes these metabolites potential targets for studying and gaining a deeper understanding of how AD progression impacts brain physiology.

## Supplemental Material

sj-docx-1-alz-10.1177_13872877251389924 - Supplemental material for Integrative analysis of cerebrospinal fluid biomarkers, metabolomics, and polygenic risk reveals novel metabolite associations with Alzheimer's disease Supplemental material, sj-docx-1-alz-10.1177_13872877251389924 for Integrative analysis of cerebrospinal fluid biomarkers, metabolomics, and polygenic risk reveals novel metabolite associations with Alzheimer's disease by Marcelo Francia, Naren Ramesh, Toni Boltz, Merel Bot, Wiesje M van der Flier, Pieter Jelle Visser, Sven van der Lee, Charlotte E Teunissen, Yolande AL Pijnenburg, Anouk den Braber, Loes Olde Loohuis, Lianne M Reus, Betty M Tijms, and Roel A Ophoff in Journal of Alzheimer's Disease

sj-zip-2-alz-10.1177_13872877251389924 - Supplemental material for Integrative analysis of cerebrospinal fluid biomarkers, metabolomics, and polygenic risk reveals novel metabolite associations with Alzheimer's disease Supplemental material, sj-zip-2-alz-10.1177_13872877251389924 for Integrative analysis of cerebrospinal fluid biomarkers, metabolomics, and polygenic risk reveals novel metabolite associations with Alzheimer's disease by Marcelo Francia, Naren Ramesh, Toni Boltz, Merel Bot, Wiesje M van der Flier, Pieter Jelle Visser, Sven van der Lee, Charlotte E Teunissen, Yolande AL Pijnenburg, Anouk den Braber, Loes Olde Loohuis, Lianne M Reus, Betty M Tijms, and Roel A Ophoff in Journal of Alzheimer's Disease
