# Supplementary material for: Integrative analysis of cerebrospinal fluid biomarkers, metabolomics, and polygenic risk reveals novel metabolite associations with Alzheimer's disease
Source: J Alzheimers Dis. 2025 Nov 7;108(4):1677–95. doi: 10.1177/13872877251389924 (PMC13247332; doi:10.1177/13872877251389924)
Supplement: sj-docx-1-alz-10.1177_13872877251389924 - Supplemental material for Integrative analysis of cerebrospinal fluid biomarkers, metabolomics, and polygenic risk reveals novel metabolite associations with Alzheimer's disease [file sj-docx-1-alz-10.1177_13872877251389924.docx]

**Supplemental Material**

**Integrative analysis of cerebrospinal fluid biomarkers, metabolomics, and polygenic risk reveals novel metabolite associations with Alzheimer’s disease**

**Supplemental files description**

Metabolomics_meta_data: Excel sheet with meta data information for all the cerebrospinal fluid (CSF) metabolites measured for this study. Unannotated metabolites measured with CSH-QTOF or HILIC-QTOF MS/MS (liquid mass spectrometry) have the following structure: "[retention time] [first five digits m/z]”. **InChI.Key:** International Chemical Identifier key. Denotes the exact stereochemical and atomic description of chemicals and used as universal identifier in chemical databases; **m.z:** mass-to-charge ratio. In metabolomics, ions are almost exclusively detected as singly charged species; **quant.mz (only available for GC-TOF):** the m/z value that was used to quantify the peak height of a BinBase entry (for GC-TOF); **RT:** retention time (seconds), **ret.index (only available for GC-TOF):** retention index, a conversion of absolute retention times to relative retention times based on a set of predefined internal standards. Classically, Kovats retention indices are used based on hydrocarbons. We use Fiehn retention indices based on FAME istd because FAME mass spectra are much easier to correctly annotate in automatic assays; **mass.spec (only available for GC-TOF):** the complete mass spectrum of the metabolite given as mz: intensity values, separated by spaces; **MSI.Level (only available for HILIC-QTOF):** the confidence level for a specific annotation, following the guidelines of the Metabolomics Standards Initiative (MSI) with slight modifications, where 1 is the highest confidence and 5 is the lowest. For 1, matching was done by m/z, retention time and MS/MS, for 2 this was m/z and MS/MS for 3 this was m/z and retention time, for 4 there is only class information available and for 5 no annotation information is available; **ESI.mode:** electrospray ionization. The method uses either negative ESI or positive ESI for negatively charged or positively charged molecules; **Species:** designates which adduct(s) are present for the annotated feature; **IUPACName:** key for the International Union of Pure and Applied Chemistry, **HMDB:** key for the The Human Metabolome Database (HMDB), **MetaboAnalystHit:** key for MetaboAnalystHi (https://www.metaboanalyst.ca), **PubChem:** public database of chemicals and chemical information

corrected-ab-metab-correlations.csv: csv file containing correlation results after correcting for diagnosis, age and gender between CSF metabolites and β-amyloid1-42 CSF levels.

corrected-ptau-metab-correlations.csv: csv file containing summary of correlation results after correcting for diagnosis, age and gender between CSF metabolites and phosphorylated Tau CSF levels.

corrected-ttau-metab-correlations.csv: csv file containing summary of correlation results after correcting for diagnosis, age and gender between CSF metabolites and total Tau CSF levels.

uncorrected-ab_corr_results.csv: csv file containing correlation results after only correcting for age and gender between CSF metabolites and β-amyloid1-42 CSF levels.

uncorrected-PTau_corr_results.csv: csv file containing summary of correlation results after only correcting for age and gender between CSF metabolites and phosphorylated Tau CSF levels.

uncorrected-TTau_corr_results.csv: csv file containing summary of correlation results after only correcting for age and gender between CSF metabolites and total Tau CSF levels.

PRS_lm_ADcohort_results.csv: csv file containing summary of linear models results using the APOE-weighted AD PRS, *APOE* ε4 and *APOE* ε2 allele counts, and the other tested traits PRS, including age and sex as cofactors, to predict CSF metabolites levels in the memory clinic cohort.

Older_PRS_lm_ADcohort_results.csv: file containing summary of linear models results using the APOE-weighted AD PRS, and the other tested traits PRS, including age and sex as cofactors, to predict CSF metabolites levels in the memory clinic cohort after stratifying by age, only including those 60 years old or older.

Younger_PRS_lm_ADcohort_results.csv: file containing summary of linear models results using the APOE-weighted AD PRS, and the other tested traits PRS, including age and sex as cofactors, to predict CSF metabolites levels in the memory clinic cohort after stratifying by age, only including those younger than 60 years old.

Older_PRS_lm_Cases_ADcohort_results.csv: file containing summary of linear models results using the APOE-weighted AD PRS, and the other tested traits PRS, including age and sex as cofactors, to predict CSF metabolites levels in the memory clinic cohort after stratifying by age, only including those 60 years old or older, as well as only those with either an MCI or AD diagnosis.

Older_PRS_lm_Controls_ADcohort_results.csv: file containing summary of linear models results using the APOE-weighted AD PRS, and the other tested traits PRS, including age and sex as cofactors, to predict CSF metabolites levels in the memory clinic cohort after stratifying by age, only including those 60 years old or older, as well as only those with either normal cognition or subjective cognitive decline.

PRS_lm_CognitivelyHealthyCohort_results.csv: csv file containing summary of linear models results using the APOE-weighted AD PRS, and the other tested traits PRS, including age and sex as cofactors, to predict CSF metabolites levels in the cognitively healthy cohort.

ELR_ConsistenPredictors.csv: csv file containing all the consistently identified metabolite predictors for both total Tau and phosphorylated Tau CSF levels over 1000 models, including a column for the frequency of the presence of the metabolites in the elastic net regression models.

ptau.prediction.freq.named.metabs.csv: csv file containing the prediction frequencies for the 678 identified CSF metabolites over 1000 models for phosphorylated Tau CSF levels, including a column for the frequency of the presence of the metabolites in the elastic net regression models.

ttau.prediction.freq.named.metabs.csv: csv file containing the prediction frequencies for the 678 identified CSF metabolites over 1000 models for total Tau CSF levels, including a column for the frequency of the presence of the metabolites in the elastic net regression models.

PaIRKAT__Results: Excel sheet with PaIRKAT pathway enrichment analysis for both total Tau and phosphorylated Tau CSF levels.

CrossStudyComparisons.csv: csv file containing a table comparing our results to the largest previous CSF metabolomics study that examined associations between CSF metabolites and CSF P-Tau and T-Tau levels.^1^ To ensure consistency across studies, metabolites were matched using the HMDB IDs included in the datasets when available.

**Supplemental Table 1**

**
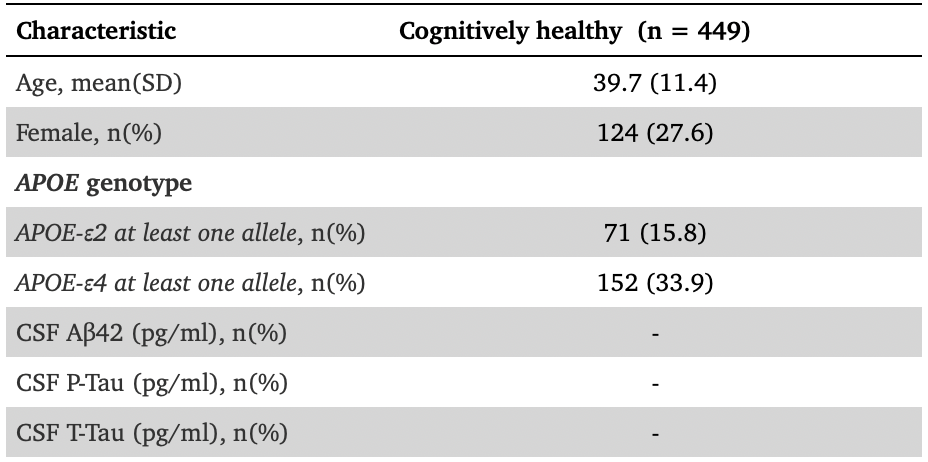
**

**Supplemental Figure 1. Distributions of AD Biomarkers Levels by Group**

(A) Distributions of CSF Amyloid Beta 42 levels for AD dementia, MCI (Mild Cognitive Impairment) and SCD (Subjective Cognitive Decline) individuals

(B) Distributions of CSF Phosphorylated Tau levels for AD dementia, MCI and SCD individuals

(C) Distributions of CSF Total Tau levels for AD dementia, MCI and SCD individuals

**
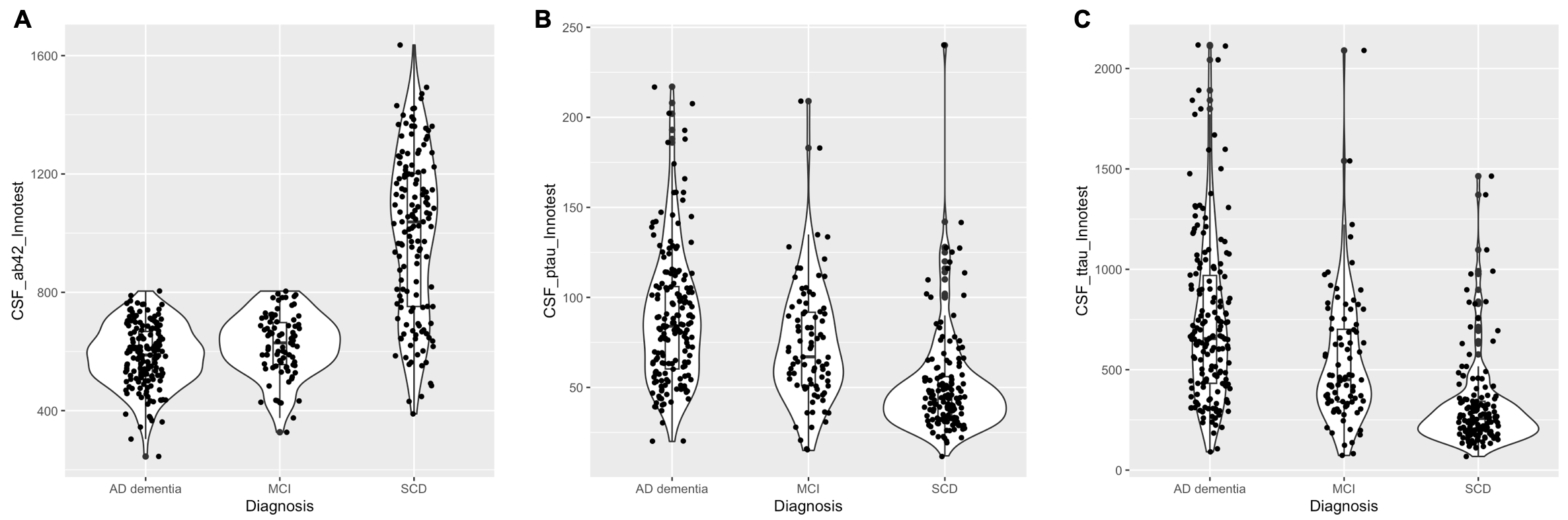
**

**Supplemental Figure 2. Comparison of significant R coefficients obtained from CSF**
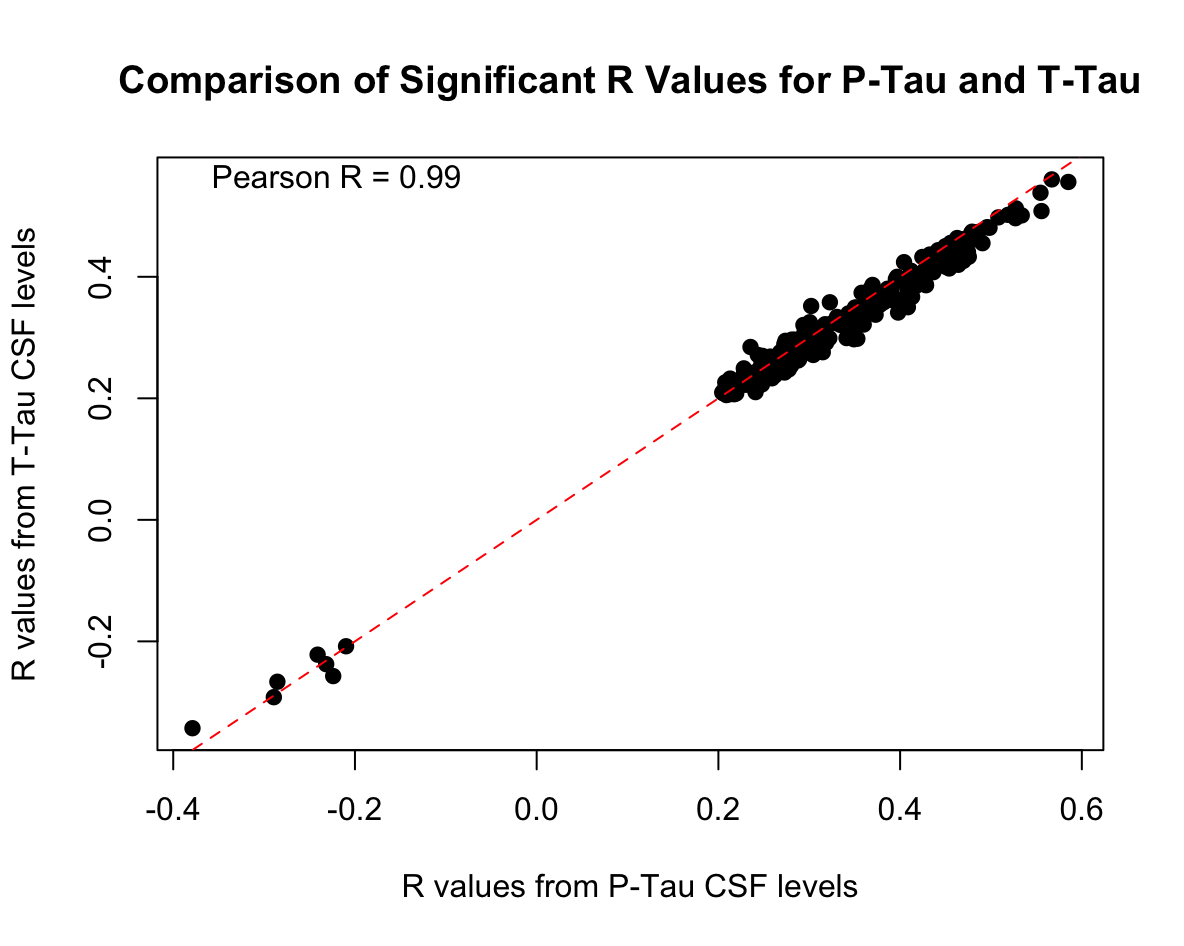
**metabolites correlations for P-Tau and T-Tau CSF levels**

Comparison of significant R coefficient obtained between CSF metabolites and either P-Tau (X axis) or T-Tau (Y axis) CSF levels. Red dotted line indicates slope of 1.

**Supplemental Figure 3. CSF metabolites and AD CSF Biomarkers correlations stratified by cofactors**

Stratification of CSF metabolites and AD CSF biomarkers total tau and phosphorylated tau, by Case/Control status. Pearson R is included inside the figure. Red and blue colors represent those metabolites found to be significantly positively or negatively correlated in the whole clinical cohort.

(A) & (B) shows only the significant CSF metabolites correlations for CSF Total Tau and P-Tau respectively

(C) & (D) shows all the non-significant CSF metabolites correlations for CSF Total Tau and P-Tau respectively.


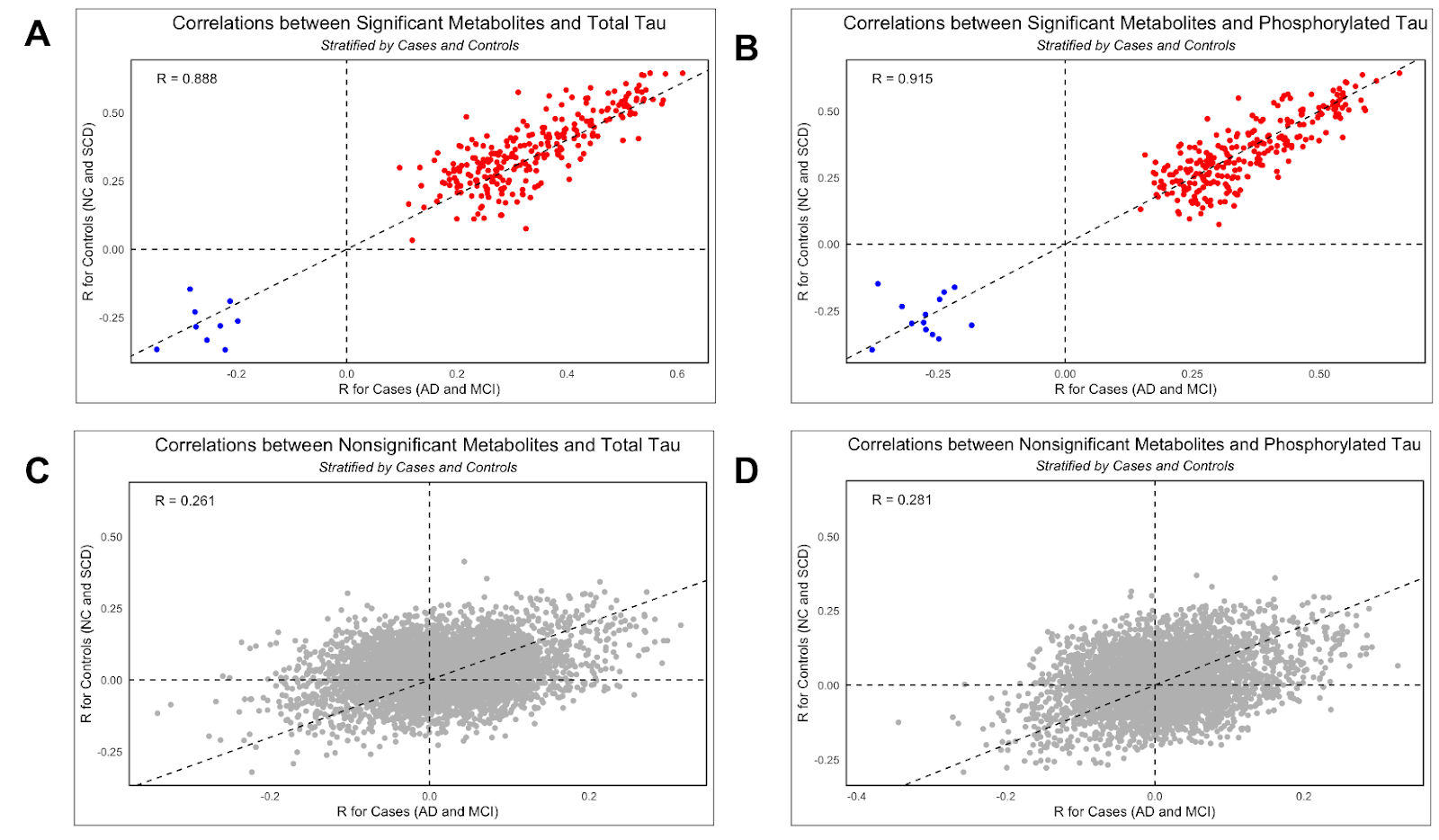


**Supplemental Figure 4. Elastic net regression results for Aβ CSF levels**

Elastic net prediction results of Aβ CSF levels using only age and sex, all the CSF metabolites (n = 5,261), and a combined model of both. Y-axis represents R values for the models. * indicates p-value below 0.01.


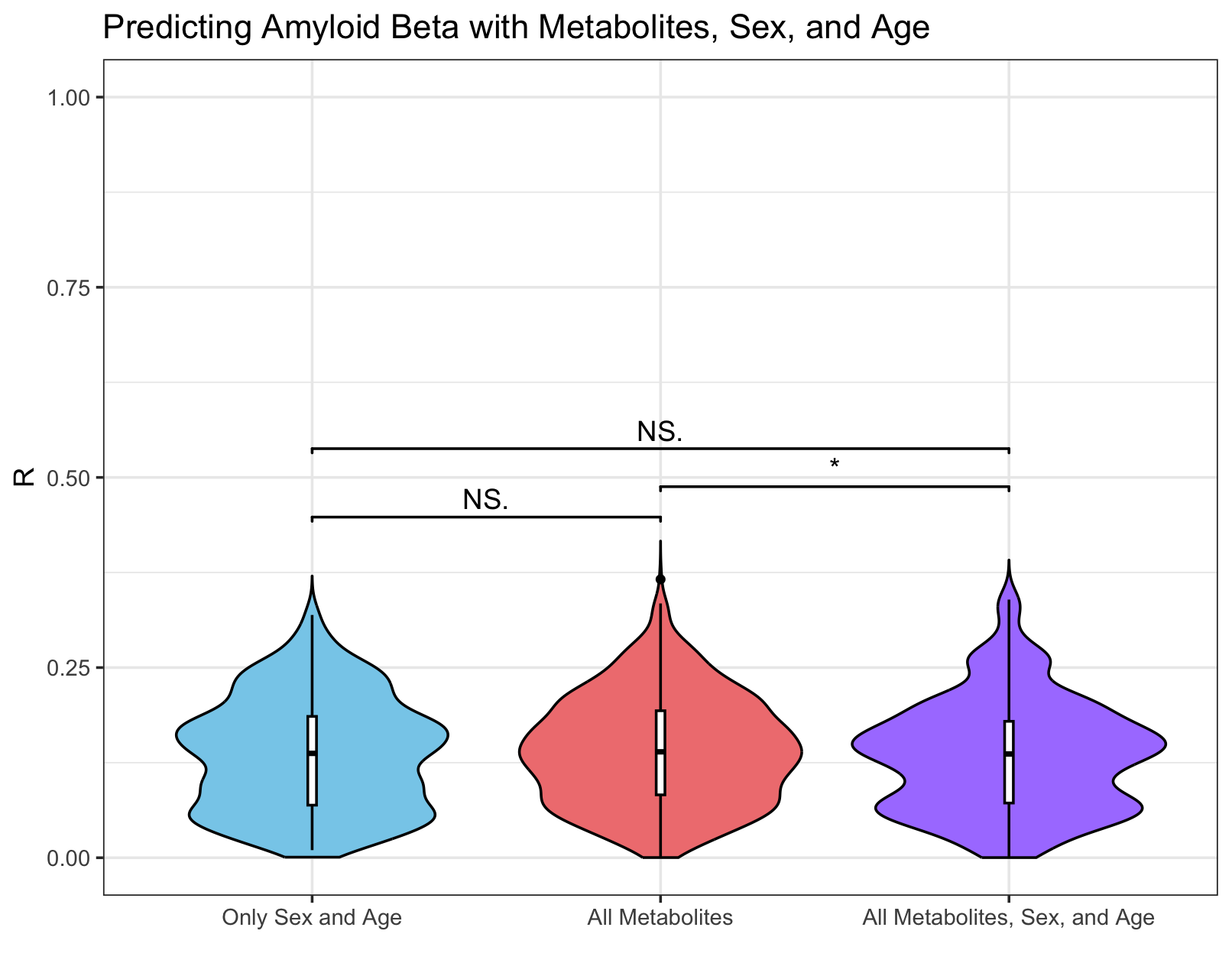


**Supplemental Figure 5. Elastic net regression results for identified CSF metabolites levels**

Elastic net prediction results of PTau (A) and TTau (B) CSF levels using all the CSF metabolites (n = 5,261), only unidentified CSF compounds (n = 4,583) and only identified CSF metabolites (n
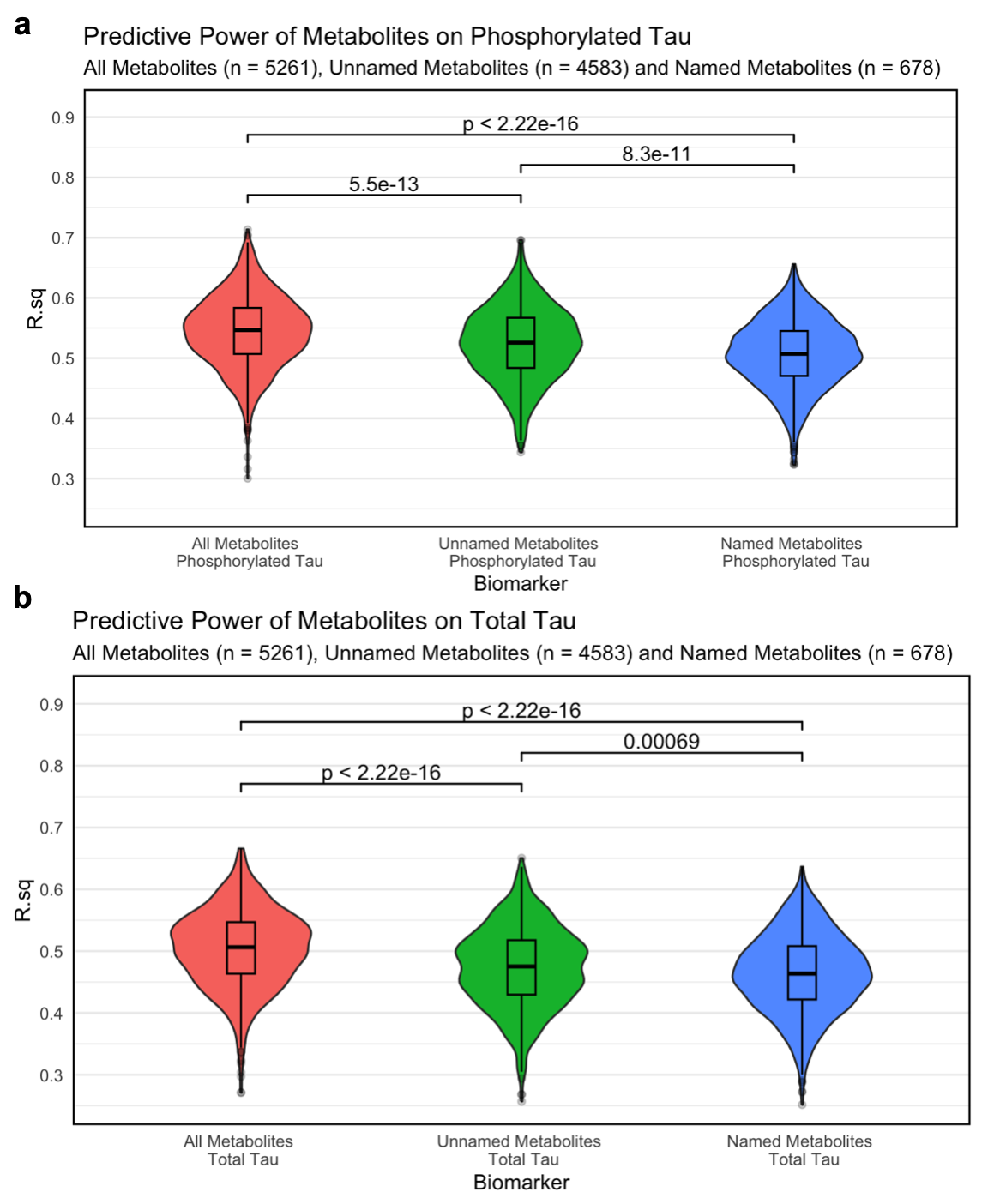
= 678). Y-axis represents R squared values for the models.

**Supplemental Figure 6. Pathway enrichment analysis results by MetaboAnalyst**

Metabolite pathway enrichment analysis results by MetaboAnalyst for CSF metabolites correlated with both CSF P-Tau and T-Tau levels (N = 288). Y-axis shows enriched metabolic pathways, x-axis indicates -log10 of the p-value. Color indicates p-value of the enrichment.


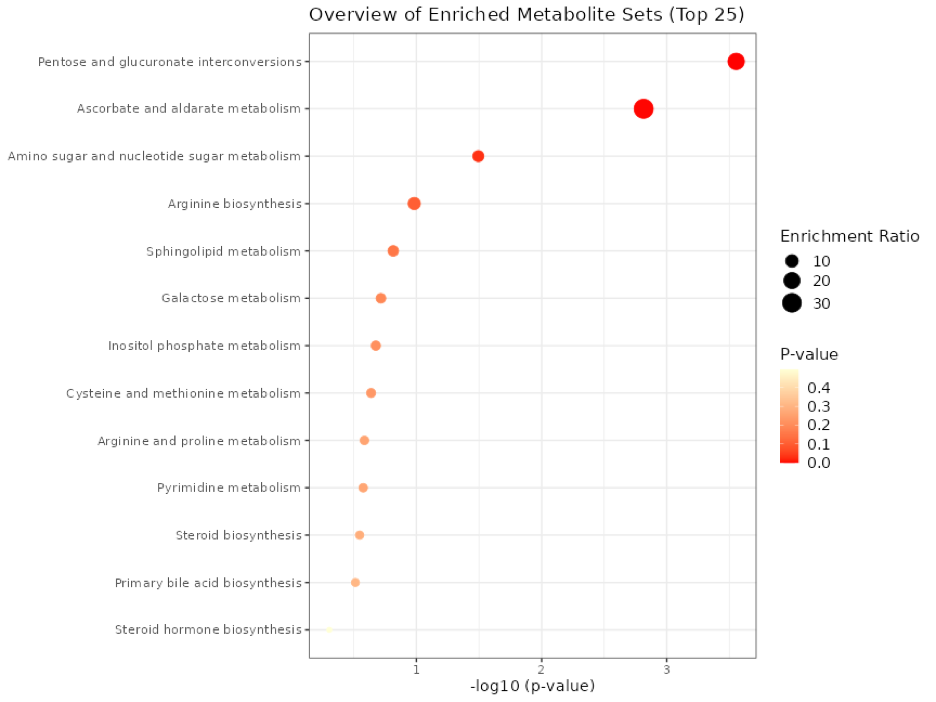


**Supplemental Figure 7. Effect of *APOE* alleles on the AD PRS**

(A) & (B) show the distribution of the AD polygenic risk scores across *APOE* ε4 allele counts, before and after removing the *APOE* locus region (Chr19: 44.4-46.5 mB).

(C) & (D) show the distribution of the AD scores across *APOE* ε2 allele counts, before and after removing the *APOE* locus.


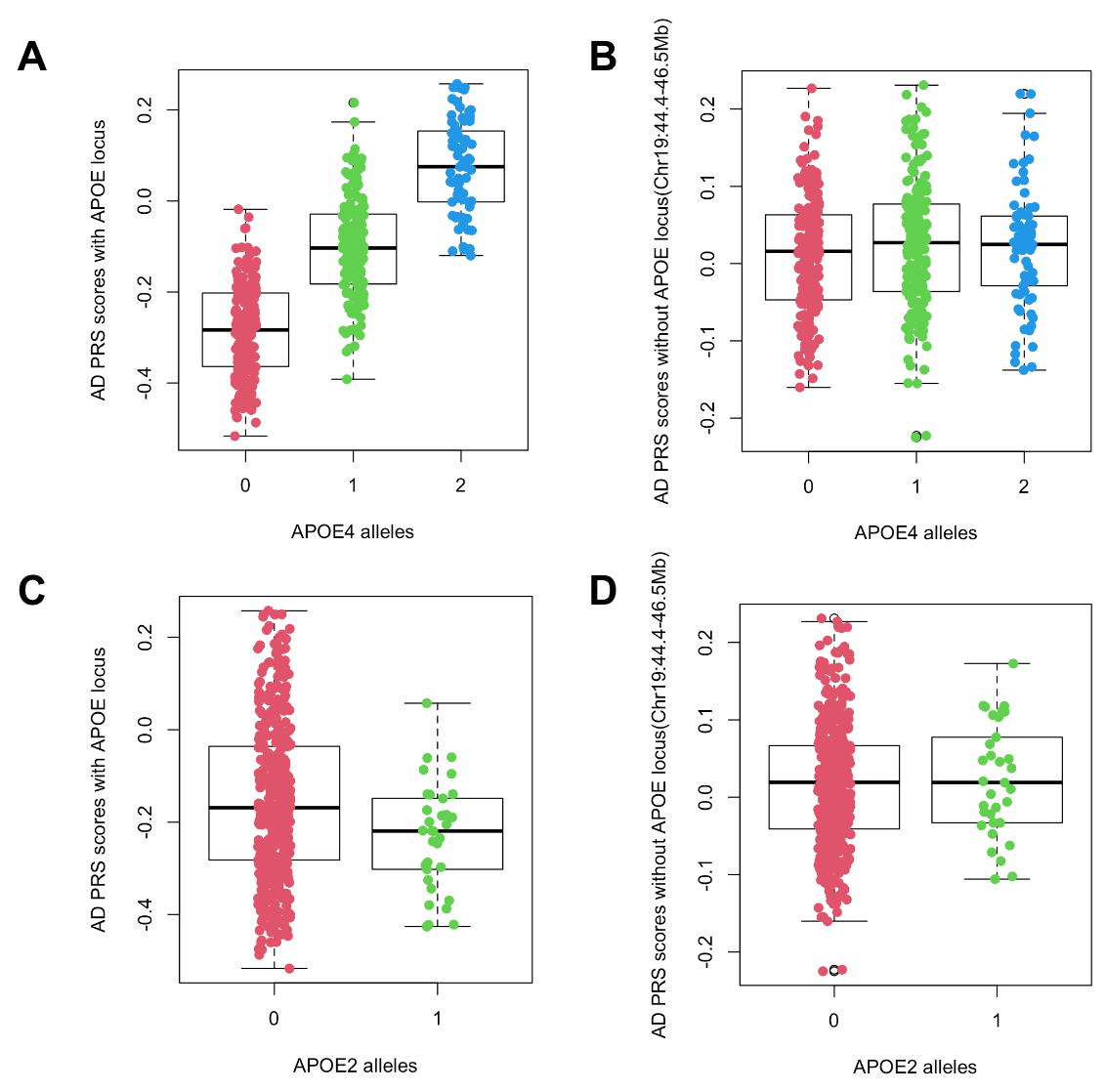


**Supplemental Figure 8. Associations between CSF metabolites and Polygenic Scores**

Linear regression analysis results for polygenic scores and CSF metabolites (n = 5,543). Y axis represents -log p value of the CSF metabolites and polygenic score association. Each dot represents a metabolite. Red line indicates Bonferroni adjusted P-value (alpha = 0.05). ADHD: attention-deficit/hyperactivity disorder; AUDI: Alcohol Use Disorder Identification Test; BPD: bipolar disorder; AD: Alzheimer’s disease; SCZ: schizophrenia

(A) Results for and the clinical cohort

(B) Results for the cognitively healthy cohort

A


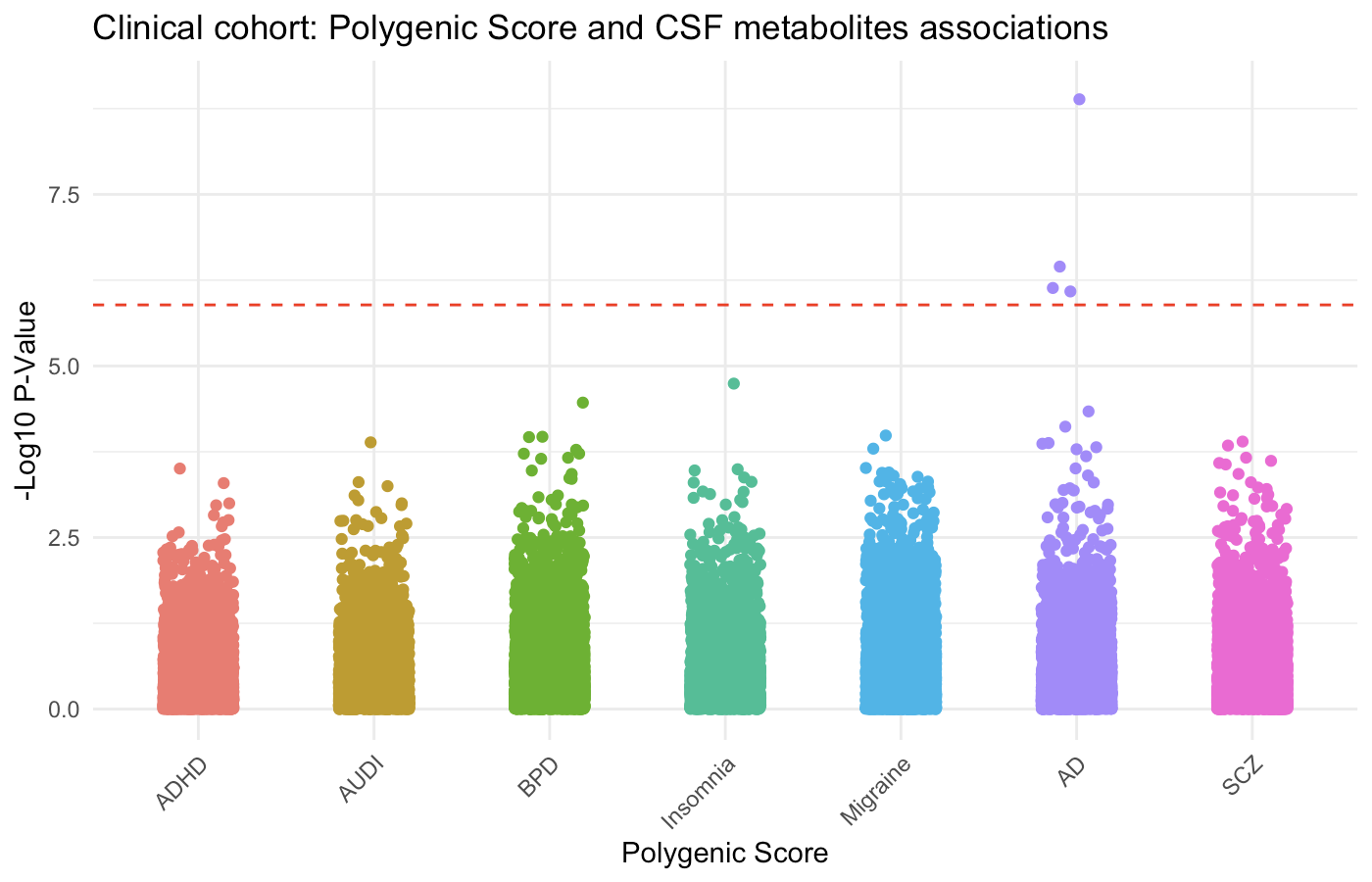


B


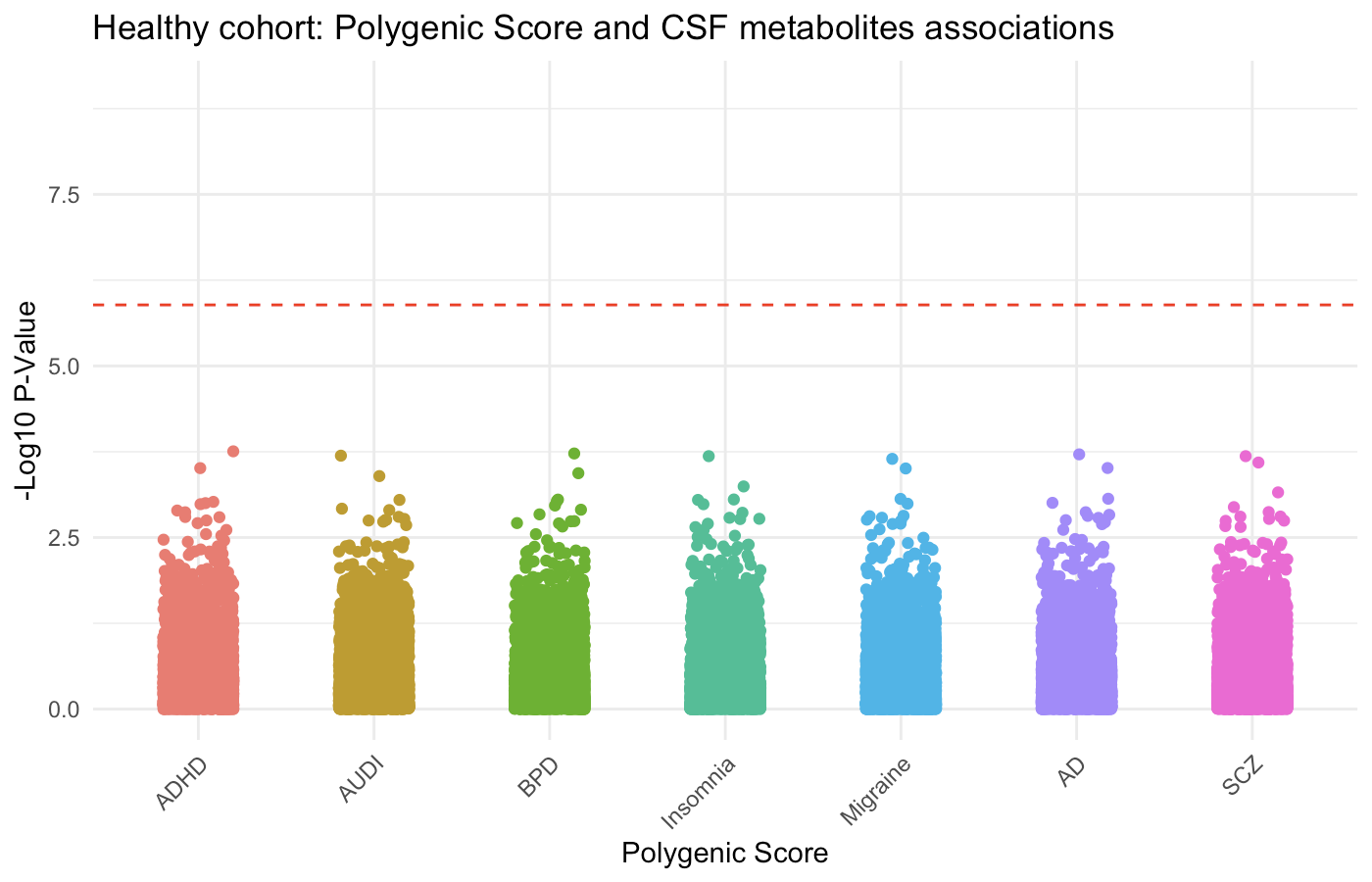


**References**

1. [Dong R, Darst BF, Deming Y, et al. CSF metabolites associate with CSF tau and improve prediction of Alzheimer’s disease status. Alzheimers Dement 2021; 13: e12167.](http://paperpile.com/b/7orT6N/4oDVK)
